# Diversity of the Paedomorphic Snail-Eating Click-Beetle Genus *Malacogaster* Bassi, 1834 (Elateridae: Agrypninae: Drilini) in the Mediterranean

**DOI:** 10.3390/biology11101503

**Published:** 2022-10-13

**Authors:** Johana Hoffmannova, Robin Kundrata

**Affiliations:** Department of Zoology, Faculty of Science, Palacky University, 17. listopadu 50, 77900 Olomouc, Czech Republic

**Keywords:** Africa, Cantharoidea, Coleoptera, Elateroidea, Italy, identification key, neoteny, Spain, systematics

## Abstract

**Simple Summary:**

The genus *Malacogaster* Bassi, 1834 belongs to the soft-bodied click-beetle tribe Drilini which contain species with a strong sexual dimorphism—while males are fully winged and able to fly, females are wingless and remain larviform. *Malacogaster* is known from the Mediterranean region, ranging from the Canary Islands and Iberian Peninsula on the west to Sicily and Libya on the east. In this study, we collated for the first time all information on this enigmatic click-beetle genus and all its species. We provide figures for all available name-bearing type specimens, redescribe species, and discuss their morphology, variability, and distribution. Although several species are readily recognizable based on the morphology and coloration, limits of some other species need further investigation including the DNA-based approach.

**Abstract:**

The soft-bodied click-beetle genus *Malacogaster* Bassi, 1834 from the Mediterranean region has never been taxonomically revised to date. Information on its morphology, intra- and interspecific variability, systematics and distribution is fragmented and most species have not been properly studied since their description. Therefore, in this study we summarize all available information on the genus *Malacogaster*. Altogether, we recognize 10 valid species from the area including the Canary Islands, Iberian Peninsula, Balearic Islands, northern coast of Africa, Sardinia, and Sicily. *Malacogaster ruficollis* Dodero, 1925, stat. nov., which was originally described as a variety of *M. bassii* Lucas, 1870 and later synonymized with it, is considered a separate species. *Malacogaster parallelocollis* Reitter, 1894, syn. nov. and *M. olcesei* var. *reductus* Pic, 1951, syn. nov. are synonymized with *M. maculiventris* Reitter, 1894. *Malacogaster notativentris* Pic, 1951, syn. nov. and *M. olcesei* Pic, 1951, syn. nov. are synonymized with *M. passerinii* Bassi, 1834. Lectotypes are designated for *M. maculiventris* Reitter, 1894, *M. nigripes heydeni* Reitter, 1894, *M. parallelocollis* Reitter, 1894, *M. thoracica* Redtenbacher, 1858, *M. olcesei* Pic, 1951, and *M. rubripes* Peyerimhoff, 1949 to fix their identity.

## 1. Introduction

**Figure 1:**
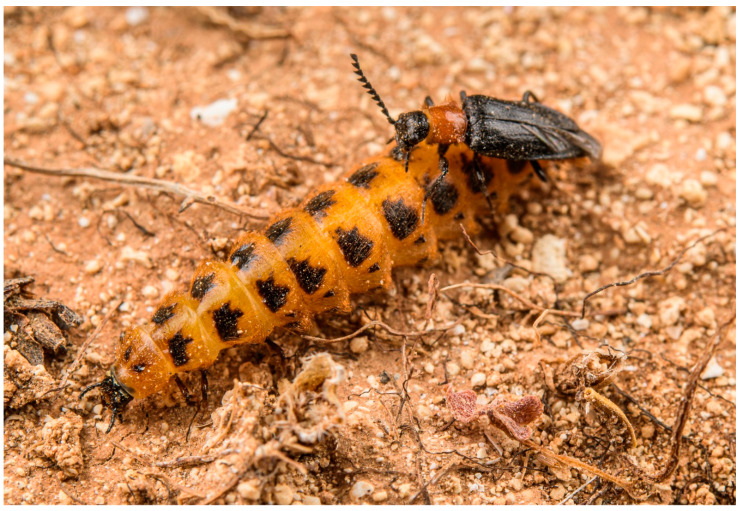
Mating couple of *Malacogaster* sp. (cf. *passerinii*) in Cala Galera on the island of Lampedusa (14 April 2018). Photograph courtesy of E. Biggi (www.anura.it (accessed on 5 April 2022)).

**Figure 2:**
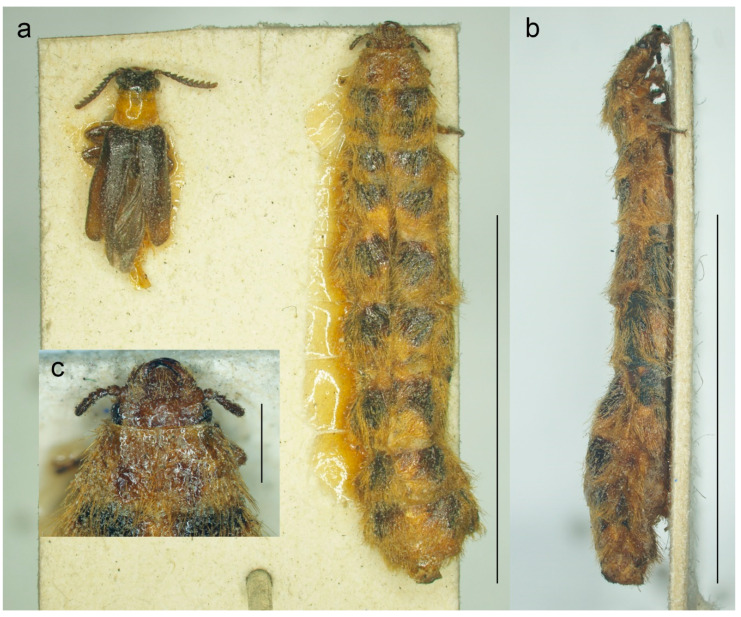
*Malacogaster passerinii* Bassi, 1834 from Sassari, Sardinia, Italy (MNHN). (**a**) Male and female habitus, dorsal view; (**b**) female habitus, lateral view; (**c**) female head, dorsal view. Scale bars = (**a**,**b**) 10.0 mm; (**c**) 1.0 mm.

The click-beetle tribe Drilini currently consists of about 150 species classified in 15 genera [1,2]. All representatives of this group are soft-bodied and affected by paedemorphic syndrome, with males being able to fly but females being larviform and completeley wingless [1,3] (Figure 1 and Figure 2a). The larvae are predators of land snails of the family Helicidae [4,5]. The history of Drilini systematic placement and classification is full of dramatic changes. Because of their soft body, they were usually placed in Malacodermata or Cantharoidea, either in Cantharidae [6,7] or in a separate family Drilidae [3,8,9], and have only relatively recently been identified as morphologically modified click-beetles of the subfamily Agrypninae [10,11,12]. The original concept of Drilidae [8,9] included many unrelated genera which were later removed from the group by Crowson [3], who kept only *Drilus* Olivier, 1790, *Malacogaster* Bassi, 1834 and *Selasia* Laporte, 1838, keeping open the possibility that a few more smaller genera might belong there. Recently, Kundrata and Bocak [1,13] described additional 10 genera from the Afrotropical Region and one genus from Pakistan, and Kovalev et al. [2] described an additional genus from Iran, increasing the number of genera in Drilini to 15.

The genus *Malacogaster* has always been a member of Drilini regardless of their concept [1,3,8,9,14,15], and its close affinities to the type genus *Drilus* have been repeatedly supported using both morphology [16] and molecular-based analyses [1,11,17]. The history of *Malacogaster* research dates back to 1834, when Bassi [18] described *Malacogaster* with its type species *M. passerinii* Bassi, 1834 from Sicily. It was the only species in the genus until Chevrolat [19] described the second species, *M. adustus* [sic!] Chevrolat, 1854, from the Levant. Redtenbacher [20] formally described *M. thoracica* which was previously known as *Ctenidion thoracicum* Dejean, 1833 [21]. Wollaston [22] added the third species, *M. tilloides* Wollaston, 1864, from the Canary Islands. Schaufuss [23] added *M. nigripes* from Spain. Lucas [24,25] published information about the *Malacogaster* larvae and described *M. bassii* from Algeria based on both sexes. Baudi di Selve [26] studied the beetle fauna of Cyprus and described, among other taxa, *M. rufipes* Baudi di Selve, 1871 and *M. truquii* Baudi di Selve, 1871.

Reitter [6] constructed an identification key to Drilini, including all then-known species of *Malacogaster*. He also identified two new species, i.e., *M. maculiventris* Reitter, 1894 from Spain and *M. parallelocollis* Reitter, 1894 from Morocco, and one new variety, i.e., *M. nigripes* var. *heydeni* Reitter, 1894 from Algeria and Morocco. Fairmaire [27] added *M. akbesiana* from the northern Levant. Olivier [8] published the first catalogue of Drilidae, including eight species of *Malacogaster* but omitting two species previously described by Reitter [6]. Zurcher [28] transfered two species from Cyprus to a related genus *Drilus*. Dodero [29] reported *M. bassii* var. *ruficollis* Dodero, 1925 from Libya. Cros [4,30] provided information on the biology and larval stages of *Malacogaster*, and reported the small differences between the most widespread species *M. nigripes* and *M. passerinii*. In his catalogue, Winkler [14] listed 10 species, ignoring the transfer of Cypriot species by Zurcher [28] to *Drilus*.

Wittmer [9] compiled a catalogue of all genera and species of the then-known Drilidae, and listed six species under *Malacogaster*, accepting the taxonomic acts by Zurcher [28] but omitting two species described by Reitter [6], probably following the catalogue of Olivier [8]. Later, Pardo Alcaide [31] and Peyerimhoff [32] described another three species from Morocco, i.e., *M. rutllanti* Pardo Alcaide, 1945, *M. holomelas* Peyerimhoff, 1949 and *M. rubripes* Peyerimhoff, 1949. Pic [33] reviewed *Malacogaster* based mainly on his material from northern Africa, and described *M. olcesei* Pic, 1941, *M. olcesei* var. *reductus* Pic, 1951, *M. theryi* Pic, 1951, *M. notativentris* Pic, 1951, *M. longicornis* Pic, 1951, and *M. curticornis* Pic, 1951. Kocher [34] catalogued the beetle fauna of Morocco and listed six species of *Malacogaster*. He made several synonymizations without any explanation; for example, he put *M. theryi* under *M. olcesei*, and *M. rutllanti* under *M. parallelocollis*.

Bahillo de la Puebla and Lopéz Colón [35] reviewed the Drilini of the Iberian Peninsula and the Balearic Islands, and summarized basic information on *M. passerinii*, *M. nigripes* and *M. maculiventris* in that region. They also provided an identification key which followed the earlier authors [30]. Bocak [15] listed 10 species in the Catalogue of the Palaearctic Coleoptera but omitted all taxa described by Pic [33]. Pic’s taxa were added later in Errata by Löbl and Smetana [36]. Kundrata and Bocak [37] provided an identification key to genera of Drilini, including *Malacogaster*. Faucheux and colleagues then published a series of descriptive papers on the morphology (mainly the antennal sensilla, mouthparts, etc.) of all stages and both sexes of *Malacogaster* from Morocco [16,38,39,40,41,42,43,44,45,46,47,48,49,50,51,52,53,54,55]. Zapata de la Vega and Sánchez-Ruiz [56] published a catalogue of Coleoptera of the Iberian Peninsula and the Balearic Islands, and listed *M. passerinii*, *M. nigripes* and *M. maculiventris*, including their distributional maps. Kundrata et al. [57] transferred the Levantine species *M. adusta* and *M. akbesiana* to genus *Drilus*. In the most comprehensive phylogenetic analysis of Drilini to date, Kundrata and Bocak [1] included two species of *Malacogaster*, tentatively redescribed the genus, and listed 11 species. Most recently, Valcárcel and Prieto Piloña [58] reported *M. nigripes* for the first time from Portugal.

The information about taxonomy, distribution and morphology of *Malacogaster* is fragmented, and most species have not been properly studied since their, often brief, description. Therefore, in this study we summarize all available information on species in the genus *Malacogaster*. This is the first attempt to study the available type specimens of *Malacogaster* species along with various other non-type specimens to understand the natural classification of the genus, including the intra- and interspecific variability. We believe that our study will serve as the first step towards understanding the diversity and species limits in *Malacogaster*, and will provide the framework for future molecular-based research.

## 2. Materials and Methods

This study is based on adult males. The genitalia were dissected after a short treatment in hot 10% KOH. The main diagnostic characters were photographed using a digital camera attached to a stereoscopic microscope. Stacks of photographs were combined with the software Helicon Focus Pro (version 7.6.4, Kharkiv, Ukraine), applying the ‘depth map’ or ‘weighted average’ rendering methods. Type specimens were examined unless stated otherwise under the respective species. Altogether, almost 150 specimens were examined. The following measurements were obtained with a scale bar in an eyepiece: body length, measured from the fore margin of head to the apex of elytra (note that the abdomen of most Drilini is highly flexible so it would be highly impractical to measure the body length to the apex of abdomen); head width including eyes; elytral length; body width, measured at humeri; pronotal length at midline; pronotal width at anterior, middle and posterior part; minimum interocular distance in the frontal part of cranium; and maximum eye diameter in the lateral view. We used the term “median antennomeres” for antennomeres V–VIII, which are usually subequal in length. We follow the morphological terminology and the definition of *Malacogaster* by Kundrata and Bocak [1]. Terminology of hind wing venation follows Lawrence et al. [59]. Label data are cited verbatim, with different lines on a label separated by a slash “/”, and different labels separated by a double slash “//”. For several species which were described based on an unknown number of specimens, we provide here the lectotype designations to fix the species identity (see Article 74 and Recommendation 73F of the Code) [60]. Publication dates of some old studies were taken from Bouchard et al. [61] and Bousquet [62]. The ZooBank LSID number for this publication is: urn:lsid:zoobank.org:pub:01E99E8D-B8CA-4A2D-93E4-FB106CF93DD7.

Abbreviations for museums and collections:
BMNHNatural History Museum, London, The United KingdomHNHMNatural History Museum, Budapest, HungaryNHMBNaturhistorisches Museum, Basel, SwitzerlandMFNBMuseum für Naturkunde Berlin, Leibniz-Institut für Evolutions- und Biodiversitätsforschung, Berlin, GermanyMHNLMusée des Confluences, Lyon, FranceMNCNMuseo Nacional de Ciencias Naturales, Madrid, SpainMNHNMuséum National d’Histoire Naturelle, Paris, FranceMSNGMuseo Civico di Storia Naturale, Genova, ItalyMUNAMuseo de Naturaleza y Arqueología, Museo de Ciencias Naturales de Tenerife, Santa Cruz de Tenerife, SpainMZLULund Museum of Zoology, Lund University, SwedenNHMWNaturhistorisches Museum, Vienna, AustriaNKMENaturkundemuseum Erfurt, GermanyNMPCNárodní muzeum, Prague, Czech RepublicOUMNHOxford University Museum of Natural History, Oxford, The United KingdomPCALprivate collection of A. Link, Ansfelden, AustriaPCATprivate collection of A. Teunissen, Eindhoven, The NetherlandsPCFHprivate collection of F. Houška, České Budějovice, Czech RepublicPCHLprivate collection of H. López, La Laguna, Tenerife, Canary Islands, SpainPCPOprivate collection of P. Oromí, La Laguna, Tenerife, Canary Islands, SpainPCRGprivate collection of R. G. Becerra, S/C de La Palma, La Palma, Canary Islands, SpainPCRKprivate collection of R. Kundrata, Olomouc, Czech RepublicSDEISenckenberg Deutsches Entomologisches Institut, Müncheberg, Germany

## 3. Results

**Genus *Malacogaster* Bassi, 1834**

(Figure 1, Figure 2, Figure 3, Figure 4, Figure 5, Figure 6, Figure 7, Figure 8, Figure 9, Figure 10, Figure 11, Figure 12, Figure 13, Figure 14, Figure 15, Figure 16, Figure 17, Figure 18, Figure 19, Figure 20, Figure 21, Figure 22, Figure 23, Figure 24 and Figure 25)

*Malacogaster* Bassi, 1834: pl. 99 [18]. Type species: *Malacogaster passerinii* Bassi, 1834: pl. 99 [18], by monotypy.

*Ctenidion*: Dejean, 1833: 104 [21] [unavailable name, published without description]. See e.g., Bassi (1834: pl. 99) [18].

*Ctenidium*: Agassiz, 1846: 107 [63] [unavailable name; emendation of unavailable name *Ctenidion*].

*Melacogaster*: Chevrolat, 1854: pl. 6 [19] [unavailable name, incorrect subsequent spelling not in prevailing usage].

*Malacoguster*: Bertolini, 1874: 132 [64] [unavailable name, incorrect subsequent spelling not in prevailing usage].

*Halacogáster*: Brues et al., 1954: 565 [65] [unavailable name, incorrect subsequent spelling not in prevailing usage].

*Malacagaster*: Faucheux, 2017: 3 [53] [unavailable name, incorrect subsequent spelling not in prevailing usage].

**Figure 3:**
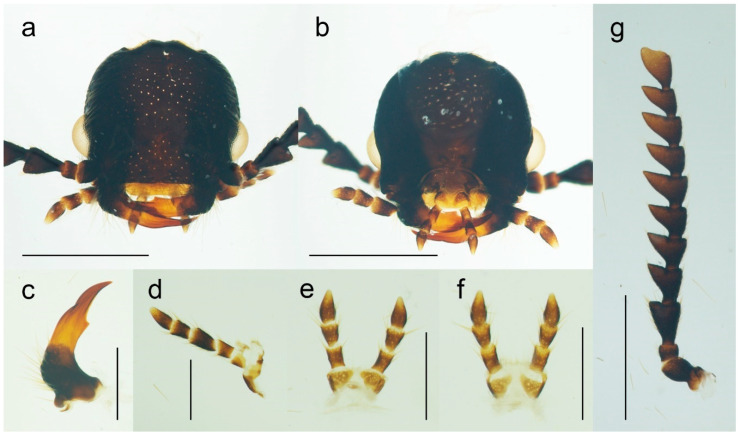
*Malacogaster passerinii* Bassi, 1834 from Sardinia, Italy, male (PCRK), details of morphology. (**a**) Head, dorsal view; (**b**) head, ventral view; (**c**) left mandible; (**d**) maxilla; (**e**) labium, ventral view; (**f**) labium, dorsal view; (**g**) right antenna. Scale bars = (**a**,**b**,**g**) 1.0 mm; (**c**–**f**) 0.5 mm.

**Figure 4:**
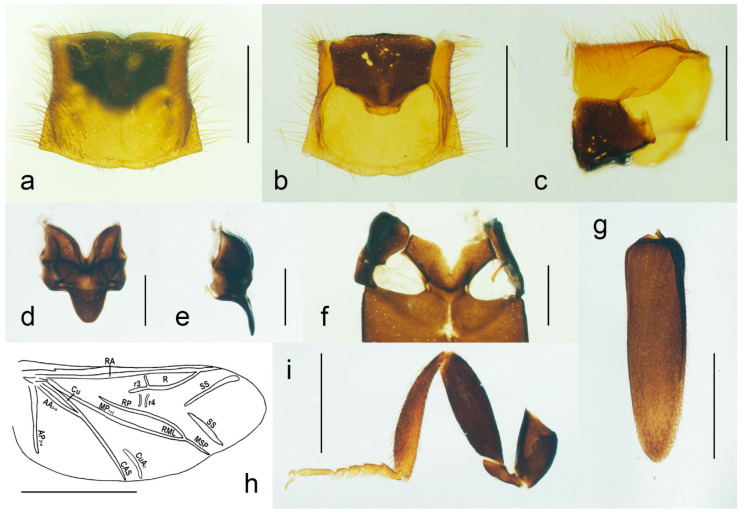
*Malacogaster passerinii* Bassi, 1834 from Sardinia, Italy (PCRK), male, details of morphology. (**a**) Prothorax dorsal view; (**b**) prothorax, ventral view; (**c**) prothorax, lateral view; (**d**) scutellum, dorsal view; (**e**) scutellum, lateral view; (**f**) mesoventrite, ventral view; (**g**) right elytron, dorsal view; (**h**) hind wing; (**i**) hind leg. Scale bars = (**a**–**c**,**i**) 1.0 mm; (**d**–**f**) 0.5 mm; (**g**) 2.0 mm; (**h**) 2.5 mm. AA3 + 4 = Anal Anterior (branches 3 + 4); AP3 + 4 = Anal Posterior (branches 3 + 4); Cu = Cubitus; CuA2 = Cubitus Anterior, branch 2; CAS = Cubitoanal Strut; MP1 + 2 = Media Posterior (branches 1 + 2); MSP = Medial Spur; R = Radial Cell; r3 = radial cross-vein 3; r4 = radial cross-vein 4; RA = Radius Anterior; RML = Radiomedial Loop; RP = Radius Posterior; SS = Support Sclerites.

**Figure 5:**
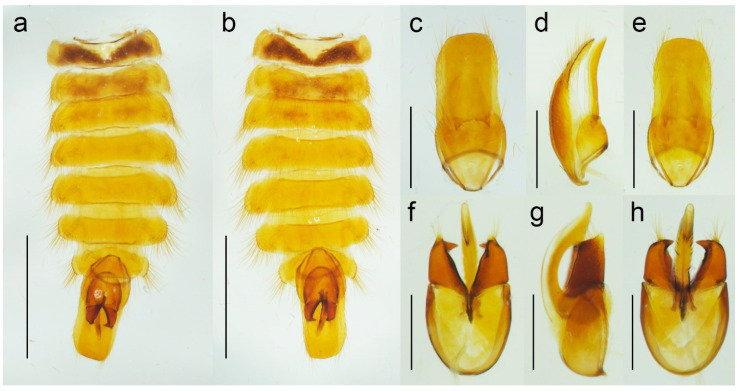
*Malacogaster passerinii* Bassi, 1834 from Sardinia, Italy (PCRK), male, details of morphology. (**a**) Abdomen, dorsal view; (**b**) abdomen, ventral view; (**c**) genital capsule formed by tergites IX and X, and sternite IX, dorsal view; (**d**) genital capsule formed by tergites IX and X, and sternite IX, lateral view; (**e**) genital capsule formed by tergites IX and X, and sternite IX, ventral view; (**f**) aedeagus, dorsal view; (**g**) aedeagus, lateral view; (**h**) aedeagus, ventral view. Scale bars = (**a**,**b**) 2.0 mm; (**c**–**e**) 1.0 mm; (**f**–**h**) 0.5 mm.

**Diagnosis**. *Malacogaster* can be recognized by the following combination of characters: antennae (Figure 3g) serrate, eyes relatively small, with their minimum frontal separation 1.85–3.00 times maximum eye diameter, mandible (Figure 3c) with only a small tooth medially at incisor, pronotum (Figure 4a,c) without sublateral carinae, lateral pronotal carina short, reaching usually no more than half the pronotal length, prosternum (Figure 4b,c) without a prosternal process, mesoventrite v-shaped, with a reduced mesoventral process, elytra (Figure 4g) usually shortened, with a rough surface, and abdomen (Figure 5a,b) with eight free ventrites.

**Redescription**. Male (Figure 1, Figure 2, Figure 8, Figure 9, Figure 10, Figure 11, Figure 12, Figure 13, Figure 14, Figure 15, Figure 16, Figure 17, Figure 18, Figure 19, Figure 20, Figure 21, Figure 22, Figure 23 and Figure 24). Body (without flexible abdomen) ca. 4.00–8.80 mm long, 2.40–3.65 times as long as wide; dark brown to black, pronotum and hypomeron usually yellowish to reddish brown (dark brown to black in *M. holomelas*; Figure 8a–c,f,g), labrum, two basal and sometimes also some apical antennomeres usually lighter than rest of antenna, some parts of abdomen and legs usually yellowish to reddish brown (whole abdomen and legs yellowish to reddish brown in *M. rutllanti*, yellowish brown in *M. rubripes*; Figure 19a–c and Figure 21c,d, respectively). Fronto-clypeal region short and wide, apically almost straight to widely concave (Figure 3a,b) (pronounced forwards and apically rounded in *M. ruficollis*); eyes small to moderate in size, their minimum frontal separation 1.85–3.00 times maximum eye diameter; labrum large, subtrapezoidal, usually well visible (transverse and hidden by anteriorly expanded fronto-clypeal region in *M*. *ruficollis*); mandible (Figure 3c) long, curved, with only small tooth medially at incisor; maxilla (Figure 3d) strongly reduced, maxillary palpus 4-segmented, with terminal palpomere apically gradually narrowed toward apex, apically usually obliquely subacute; labium (Figure 3e,f) strongly reduced, partly membranous, labial palpus 3-segmented, with terminal palpomere apically gradually narrowed toward apex, apically usually obliquely subacute; antenna (Figure 3g) serrate, with 11 antennomeres, with pedicel small, shortest, antennomeres 3–10 strongly serrate, apical antennomere simple, usually obliquely truncate (but often variously deformed). Pronotum (Figure 4a) subquadrate to subtrapezoidal, 1.00–1.35 times as wide as long when measured at widest place, widest usually posteriorly (in some cases medially or medially and posteriorly, in *M. tilloides* anteriorly), with lateral sides slightly concave, subparallel or slightly rounded, posterior margin with small arcuate median emargination; lateral carina short, reaching usually no more than half of pronotal length; prosternum (Figure 4b) more or less strongly transverse, without distinct chin-piece, anteriorly almost straight to slightly rounded, posteriorly sloping down, slightly produced medially, with reduced prosternal process; internal prothoracic processes very short; pronotosternal sutures short, simple, almost straight; scutellar shield (Figure 4d,e) on same plane as anterior part of scutellum, tongue-like, basally slightly wider than long, sides rounded, gradually narrowed toward apex, narrowly rounded to subtruncate apically; mesoventrite (Figure 4f) v-shaped, with usually only indistinctly defined shallow mesoventral cavity, anteriorly often partly membranous, mesoventral process more or less reduced; mesocoxal cavity open to both mesanepisternum and mesepimeron; metanotum roughly subquadrate, with straight and medially thickened scutoscutellar ridges, and with moderately deep median groove, postnotal plate subtrapezoidal, slightly wider than long; elytra (Figure 4g) usually relatively short compared to length of elytra in other Drilini, combined 1.55–2.55 times as long as wide, and 2.65–3.85 times as long as pronotal length, dehiscent, only partly covering abdomen, each elytron apically independently rounded, with surface uneven, without distinct striae or lines of puncture, irregularly punctured; epipleuron developed basally, then gradually distinctly narrowed, reduced after half of elytral length. Hind wing venation as in Figure 4h; cubital and medial portion reduced, CuA2 incomplete, wedge cell absent, radial cell approximately 3.7 times as long as wide, two weak support sclerites in apical portion perpendicular to each other. Legs (Figure 4i) slender, slightly compressed; tarsomeres I–IV gradually shorter, tarsomere IV with small ventral lobe, apical tarsomere longest; claws simple, curved, each basally with long seta. Abdomen (Figure 5a,b) with eight free ventrites connected by highly flexible extensive membranes; first ventrite partly reduced anteromedially; abdominal sternite IX elongate, usually 2.15–2.70 times as long as wide (1.65 times in *M. ruficollis*); abdominal tergites IX and X tightly connected by membrane; tergite X usually elongate, 1.85–2.25 times as long as wide (Figure 5c–e) (1.50 times in *M. tilloides*, and subquadrate, 0.95 times as long as wide in *M. ruficollis*). Aedeagus (Figure 5f–h) elongate, trilobate; median lobe distinctly surpassing apices of parameres, strongly curved in lateral view, with distinct subapical hook; parameres robust, shorter than phallobase, variously shaped but in most species truncate apically, with latero-apical projection on inner side (very slightly developed in *M. ruficollis*, not developed in *M. tilloides*); phallobase robust, u-shaped.

**Figure 6:**
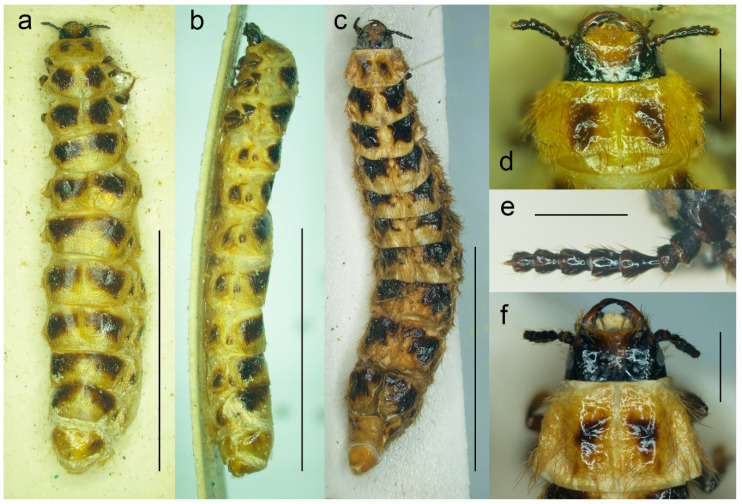
*Malacogaster* sp., female morphology. (**a**) Specimen from northern Africa (MNHN), habitus, dorsal view; (**b**) specimen from northern Africa (MNHN), habitus, lateral view; (**c**) specimen from Mallorca (MZLU), habitus dorsal view; (**d**) specimen from northern Africa (MNHN), head, dorsal view; (**e**) specimen from Mallorca (MZLU), right antenna; (**f**) specimen from Mallorca (MZLU), head, dorsal view. Scale bars = (**a**–**c**) 10.0 mm; (**d**,**f**) 1.0 mm; (**e**) 0.5 mm.

Female (Figure 2 and Figure 6). Larviform, body elongate (Figure 2a,b and Figure 6a–c), up to ca. 28 mm long and ca. 6 mm wide (usually smaller). All body parts yellowish to reddish brown; major parts of head including antennae, and legs usually dark brown, dorsal surface of thoracic and abdominal segments each with two dark brown markings, usually smaller on pronotum. Head prognathous (Figure 2c and Figure 6d,f) well sclerotized, small, including eyes narrower than pronotal anterior margin, with shallow depression between antennal sockets; clypeus short, widely concave anteriorly. Eyes small, not protruding. Antennae short (Figure 6e), with eight antennomeres, penultimate antennomere bearing small conical appendage, ultimate antennomere minute, distinctly smaller than other antennomeres. Mandibles robust, shiny, considerably curved, incisor margin with small tooth in middle part. Pronotum widest posteriorly. Legs short, robust. Abdomen with apical segment much narrower and smaller.

**Figure 7:**
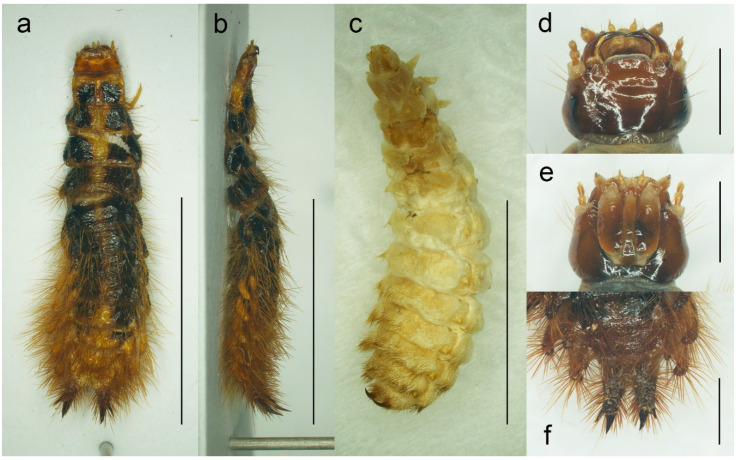
*Malacogaster* sp. from Morocco (PCRK), larval morphology. (**a**) Active larva, habitus, dorsal view; (**b**) active larva, habitus, lateral view; (**c**) pseudopupa, habitus, dorsal view; (**d**) active larva, head, dorsal view; (**e**) active larva, head, ventral view; (**f**) active larva, apex of abdomen, dorsal view. Scale bars = (**a**–**c**) 8.0 mm; (**d**,**e**) 1.0 mm; (**f**) 1.50 mm.

Active larva (Figure 7a,b,d–f). Body elongate, slightly widened towards apex. Body yellowish brown to dark reddish brown, often with head darker, smaller to very large markings on thoracic and some abdominal segments, including pleural and tergal processes, dark brown. Head prognathous, well sclerotized, small, almost as wide as frontal pronotal margin. Antennae three-segmented. Mandibles well developed, simple, narrow and falcate. Abdomen with sclerotized and pigmented tergites, with lateral tergal and pleural processes, covered with long setae, especially dorsally and more towards apex. Cerci bent slightly upward, each covered with long setae, apically narrow, sharp, with one additional subapical spine laterally. For more information, see e.g., [4,47,49].

Pseudopupa (Figure 7c). The same as active larva but more robust, much lighter, usually light yellowish to yellowish brown, with legs stouter, and hairs only on several apical abdominal segments. For more information, see e.g., [47,49].

**Composition**. 10 species: *Malacogaster bassii* Lucas, 1870, *M. holomelas* Peyerimhoff, 1949, *M. maculiventris* Reitter, 1894, *M. nigripes* Schaufuss, 1867, *M. passerinii* Bassi, 1834, *M. rubripes* Peyerimhoff, 1949, *M. ruficollis* Dodero, 1925, stat. nov., *M. rutllanti* Pardo Alcaide, 1946, *M. theryi* Pic, 1951, *M. tilloides* Wollaston, 1864.

**Biology**. For most species, there is no information on their biology. Cros [4] provided information on biology of *Malacogaster* sp. from northern Africa. More recently, Faucheux and colleagues [38,47,48,49] published a series of papers with information on biology of *Malacogaster* species from Oualidia, Morocco, which they first identified as *M. passerinii* and later changed their identification to *M. nigripes* [48]. The larvae of *Malacogaster* are known to prey on several land snail species including e.g., *Theba pisana* (Müller, 1774), *Helix* spp., *Sphincterochila candidissima* (Draparnaud, 1801), *Sphincterochila cariosula* (Michaud, 1833), and *Xerophila mauritanica* (Bourguignat in Servain, 1881) [4,35,38,39]. Females of *Malacogaster* from Morocco were observed feeding on *Cepaea hortensis* (Müller, 1774) [38]. There are no observations on feeding of the adult males.

**Distribution**. Italy (including Sardinia, Sicily, and nearby islets), Spain (including Canary Islands, Balearic Islands), Portugal, Gibraltar; northern Africa (Morocco, Algeria, Tunisia, Libya) (Figure 25).

**Literature**. Dejean (1833: 104): catalogue, *Ctenidion* [published without description] [21]; Bassi (1834: pl. 99): original description of *Malacogaster*, drawings of male dorsal and lateral habitus, head, abdomen, antenna, and leg [18]; Dejean (1837: 117): catalogue, *Ctenidion* [published without description] [66]; Westwood (1838: 246): remark [67]; Sturm (1843: 77): checklist [as *Ctenidion*] [68]; Agassiz (1846: 47, 97): checklist; *Ctenidion* and *Malacogaster*, respectively [69]; Agassiz (1846: 107, 223): checklist; *Ctenidion* and *Malacogaster*, respectively [63]; Lucas (1847: 185): catalogue [70]; Chevrolat (1854: 433/pl. 6): species description [currently in *Drilus*; also as *Melacogaster*; sic!] [19]; Rosenhauer (1856: 140): remark [71]; Lacordaire (1857: 369/371): catalogue [72]; Redtenbacher (1858: 525): catalogue, species description [20]; Desmarest (1860: 8): remark [73]; Jacquelin du Val (1860: 164): catalogue, drawing of male habitus [74]; Wollaston (1864: 215): species description [22]; Wollaston (1865: 193): catalogue [75]; Kiesenwetter (1866: 244): distributional remark [76]; Schaufuss (1867: 83): species description [also as *Melac.*; sic!] [23]; Gemminger (1869: 1684): catalogue [77]; Lucas (1870: lvii): remark, species description [24]; Gerstaecker (1870: 55): remark [78]; Baudi di Selve (1871: 61): species descriptions [currently in *Drilus*] [26]; Lucas (1871: 19): species redescription, drawings of male and female habitus [25]; Marseul (1873: 413): catalogue [79]; Redtenbacher (1873: 19): catalogue, redescription [80]; Bertolini (1874: 132): catalogue [as *Malacoguster*; sic!] [64]; Marseul (1877: 42): catalogue [81]; Oliveira (1884: 190): catalogue [82]; Scudder (1884: 84, 186): checklist; *Ctenidion* and *Malacogaster*, respectively [83]; Failla-Tedaldi (1887: 159): remark [84]; Moragues (1889: 24): catalogue [85]; Ragusa (1893: 358): catalogue [86]; Reitter (1894: 3): identification key, species descriptions [6]; Fairmaire (1895: cx): species description [currently in *Drilus*] [27]; Medina (1895: 44): catalogue [87]; Bertolini (1900: 71): catalogue [88]; Xambeu (1901: 37): larva description [89]; Bourgeois (1904: 481): remark [90]; Rosenberg (1909: 232): remark [91]; Olivier (1910: 4): catalogue [8]; Zurcher (1911: 243): taxonomic remarks [28]; Escalera (1914: 225): catalogue [92]; Peyerimhoff (1914: 268): remark [93]; Peyerimhoff (1935: 19): remark [94]; Rüschkamp (1920: 386): distribution [95]; Zanon (1922: 123): catalogue [7]; Cros (1925: 301): remarks, also on larva [30]; Dodero (1925: 7): species description [29]; Seurat (1925: 285): remark [96]; Winkler (1925: 522): catalogue [14]; Cros (1926: 183): remarks, drawing of larval abdominal apex [97]; Luigioni (1929: 616): catalogue [98]; Porta (1929: 47): catalogue [99]; Cros (1930: 133): biology, redescription, larva description, drawings of larval abdominal apex and mouthparts [4]; Gridelli (1930: 97): catalogue, remarks [100]; Fuente (1931: 64): catalogue [101]; Pic and Lindberg (1932: 3): catalogue [102]; Balduf (1935: 101): larva, biology [103]; Neave (1939: 894): checklist [as *Ctenidion*] [104]; Clausen (1940: 544): remark [105]; Neave (1940: 31): checklist [106]; Seabra (1943: 64): catalogue [107]; Wittmer (1944: 204): catalogue [9]; Pardo Alcaide (1945: 457): catalogue, species description [31]; Wittmer (1948: 115): catalogue [108]; Cobos (1949: 568): distributional remark, checklist [109]; Peyerimhoff (1949: 265): species description [32]; Pic (1951: 295): revision, species descriptions [33]; Harvey (1952: 392): remark [110]; Brues et al. (1954: 565): remark [as *Halacogáster*; sic!] [65]; Kocher (1956: 24): catalogue [34]; Goidanich (1957: 564): remark, biology [111]; Gridelli (1960: 386): catalogue [112]; Torres Sala (1962: 239): catalogue [113]; Kocher (1964: 44): catalogue [114]; Magis (1966: 459): remarks [115]; Kocher (1969: 42): catalogue [116]; Crowson (1972: 51): taxonomic remark [3]; Baronio (1974: 175): remark [117]; Israelson et al. (1982: 118): catalogue [118]; Schilthuizen et al. (1994: 184): remark [119]; Lo Valvo and Massa (1995: 883): checklist [120]; Poggi (1995: 6): checklist [121]; Sparacio (1997: 65): catalogue [122]; Machado and Oromí (2000: 53): catalogue [123]; Poggi (2003: online): catalogue [124]; Bahillo de la Puebla et al. (2004: 140): remark [125]; Bahillo de la Puebla and López Colón (2005: 124): revision, identification key, distributional map [35]; Bocak (2007: 210): catalogue [15]; Kundrata & Bocak (2007: 427): remark, identification key [37]; Bocak and Brlik (2008: 191): remark [126]; Faucheux and Agnas (2008: 109): hypermetamorphosis, larva and female morphology, distributional map [38]; Bocak et al. (2010: 104): review, drawing of basal antennomeres, photographs of larvae [127]; Löbl and Smetana (2010: 25): catalogue [36]; Oromí et al. (2010: 279): catalogue [128]; Faucheux and Agnas (2011: 79): biology of larvae and females [39]; Kundrata and Bocak (2011: 365): taxonomic remark [10]; Kundrata (2012: 261): remark [129]; Kundrata (2012: 217): remark [130]; Zapata de la Vega and Sánchez-Ruiz (2012: 125): catalogue, distributional maps [56]; Zapata de la Vega and Sánchez-Ruiz (2013: 180): catalogue [131]; Faucheux and Agnas (2014: 258): remark [132]; Faucheux and Kundrata (2014: 97): morphology of female antenna [133]; Kundrata et al. (2014: 167): molecular phylogeny [11]; Kundrata et al. (2014: 457): taxonomy [57]; Zapata de la Vega and Sánchez-Ruiz (2014: 157): catalogue [134]; Faucheux (2015: 57): remark on larva [135]; Faucheux (2015: 73): remark on female antennae and biology [136]; Faucheux (2015: 188): remark [137]; Kobieluszova and Kundrata (2015: 91): female antennal morphology [138]; Kundrata et al. (2015: 52): remark, comparison with other genus [139]; Petrzelkova and Kundrata (2015: 485): remark [this species is currently in *Drilus*] [140]; Trllova and Kundrata (2015: 563): taxonomic remark, comparison with other genus [141]; Zapata de la Vega and Sánchez-Ruiz (2015: 186): catalogue [142]; Agnas and Faucheux (2016: 180): biology, reproduction [40]; Baalbergen et al. (2016: 168): remark on larva [5]; Bocak et al. (2016: Supplementary Materials): molecular phylogeny [143]; Faucheux (2016: 165): antennal morphology [41]; Faucheux (2016: 201): hypermetamorphosis, identification problem [42]; Faucheux (2016: 221): biology and ecology [43]; Faucheux (2016: 229): antennal morphology [44]; Faucheux (2016: 267): larval remark, identification problem [144]; Faucheux (2016: 288): female antennal morphology, identification problem [45]; Faucheux (2016: 311): male antennal morphology, comparison with other species, identification problem [46]; Faucheux and Agnas (2016: 60): hypermetamorphosis [47]; Faucheux and Ballardini (2016: 187): biology, mating, breeding, identification problem [48]; Faucheux and Beaulieu (2016: 102): hypermetamorphosis [49]; Faucheux and Beaulieu (2016: 107): primary larval antennae and associated sensilla [50]; Faucheux and Beaulieu (2016: 149): male antennal sensilla [145]; Faucheaux et al. (2016: 121): mouthparts and associated sensilla in primary larva [51]; Faucheaux et al. (2016: 1): larval and adult morphology, ecology, distributional map, comparison with other species [146]; Kundrata et al. (2016: 296): molecular phylogeny [147]; Zapata de la Vega and Sánchez-Ruiz (2016: 178): catalogue [148]; Faucheux (2017: 1): female mouthparts and sensilla, biology, taxonomic remark [52]; Faucheux (2017: 17): remark on biology [149]; Faucheux (2017: 1): male mouthparts and sensilla, biology, taxonomic remark [also as *Malacagaster*; sic!] [53]; Faucheux (2017: 1): morphology of female mouthparts, taxonomic remark [150]; Faucheux and Kundrata (2017: 106): antennal morphology, taxonomic remark [16]; Kundrata and Bocak (2017: 442): taxonomic remark [13]; Zapata de la Vega and Sánchez-Ruiz (2017: 275): catalogue [151]; Faucheux (2018: 1): antennal morphology of female immature stages, remark on biology, taxonomic remark [54]; Faucheux and Agnas (2018: 1): teratology of female antennae, taxonomic remark [55]; Kundrata et al. (2018: suppl.): molecular phylogeny [152]; Lequet and Faucheux (2018: 14): remark on breeding [153]; Sormova (2018: 267): taxonomic remark [154]; Sormova et al. (2018: 1): taxonomic and distributional remark, molecular phylogeny [155]; Zapata de la Vega and Sánchez-Ruiz (2018: 31): catalogue [156]; Bi et al. (2019: 82): molecular phylogeny [157]; Bocak et al. (2019: 142): taxonomic remark [12]; Kovalev et al. (2019: 187): taxonomic remark, comparison with other genus [2]; Kundrata and Bocak (2019: 414/441): molecular phylogeny, review, comparison with other genera, photographs of male pronotum, mesoventrite and abdomen [1]; Kundrata et al. (2019: 100): generic catalogue [158]; Ortego (2019: 340): type material information [159]; Zapata de la Vega and Sánchez-Ruiz (2019: 74): catalogue [160]; Chavanon (2020: 69): catalogue [161]; Lo Valvo (2020: 170): checklist [162]; Valcárcel and Prieto Piloña (2020: 317): distribution [58]; Dal Cortivo et al. (2021: 20/69): key, checklist, biology [163]; Douglas et al. (2021: 10): molecular phylogeny [17]; Poggi (2021: online): catalogue [164].

### 3.1. Species Currently Included in Genus Malacogaster Bassi, 1834

#### 3.1.1. *Malacogaster bassii* Lucas, 1870

(Figure 25a)

*Malacogaster bassii* Lucas, 1870: lviii [24].

*Malacogaster bassi*: Reitter, 1894: 5 [6] [unavailable name, incorrect subsequent spelling not in prevailing usage].

*Malacogaster massi*: Cros, 1925: 301 [30] [unavailable name, incorrect subsequent spelling not in prevailing usage.

**Type material**. Described based on an unknown number of male and female specimens [24,25]. Type material has not been found despite a thorough search in major European museums including MNHN.

**Type localities**. Algeria: Oran and Tlemcen.

**Material examined**. None.

**Diagnosis**. Based on Lucas [25]. Male. Body length 8.50 mm, body width 5.75 mm (obviously an error). Head, antennomeres I–X, femora, tibiae, scutellum, and elytra black, antennomere XI, tarsi, and abdomen yellowish to reddish brown, pronotum reddish brown near margins, medially distinctly darker.

Female. Body 28 mm long, 6 mm wide. All body parts yellowish to reddish brown; margins of head, antennae, dorsal surface of thoracic and most abdominal segments (except apical ones) with a large black patch on each side.

**Distribution**. Algeria (Figure 25a).

**Literature**. Lucas (1870: lviii): original description [24]; Gerstaecker (1870: 55): remark [78]; Lucas (1871: 22): redescription, drawings of male and female habitus [25]; Marseul (1877: 42): catalogue [81]; Reitter (1894: 5): identification key [as *M. bassi*; sic!] [6]; Olivier (1910: 4): catalogue [8]; Cros (1925: 301): remark [as *M. massi*; sic!] [30]; Dodero (1925: 7): comparison with other species, description of a new variety [considered as a separate species in this study] [29]; Winkler (1925: 523): catalogue [14]; Cros (1926: 184): remark on female [97]; Cros (1930: 133): comparison with other species [4]; Gridelli (1930: 97): catalogue, remark [100]; Wittmer (1944: 204): catalogue [9]; Pic (1951: 295): remarks [33]; Kocher (1956: 25): taxonomic remark, synonymization with *M. passerinii* [34]; Goidanich (1957: 565): remark, as synonym with *M. passerinii* [111]; Bocak (2007: 210): catalogue [15]; Faucheaux et al. (2016: 53): remark [146]; Faucheux (2017: 14): remark [52]; Kundrata and Bocak (2019: 441): review [1].

**Remarks**. This species was not examined by earlier authors, e.g., [6,33,34], so the type material might have been lost. Additionally, no specimens other than the types have been reported to date. Based on the drawing of a male habitus by Lucas [25], *M. bassii* is similar to *M. ruficollis* in the general coloration and the pronotum which is distinctly narrowed at the anterior third just before anterior angles (Figure 20g). However, all available specimens of *M. ruficollis* are smaller, maximally up to 7.20 mm long, and are known only from the Cyrenaica region of Libya. It is more probable that *M. bassii* is in fact conspecific with either *M. nigripes* (its former var. *heydeni* from Algeria, which is generally more robust and some specimens have slightly darker parts of the pronotum, see Figure 13c) or widely delimited *M. passerinii*, which often has dark legs in northern Africa (see e.g., Figure 18g). It should be noted that Kocher [34] considered *M. bassii* and *M. passerinii* synonyms but without any explanation.

#### 3.1.2. *Malacogaster holomelas* Peyerimhoff, 1949

**Figure 8:**
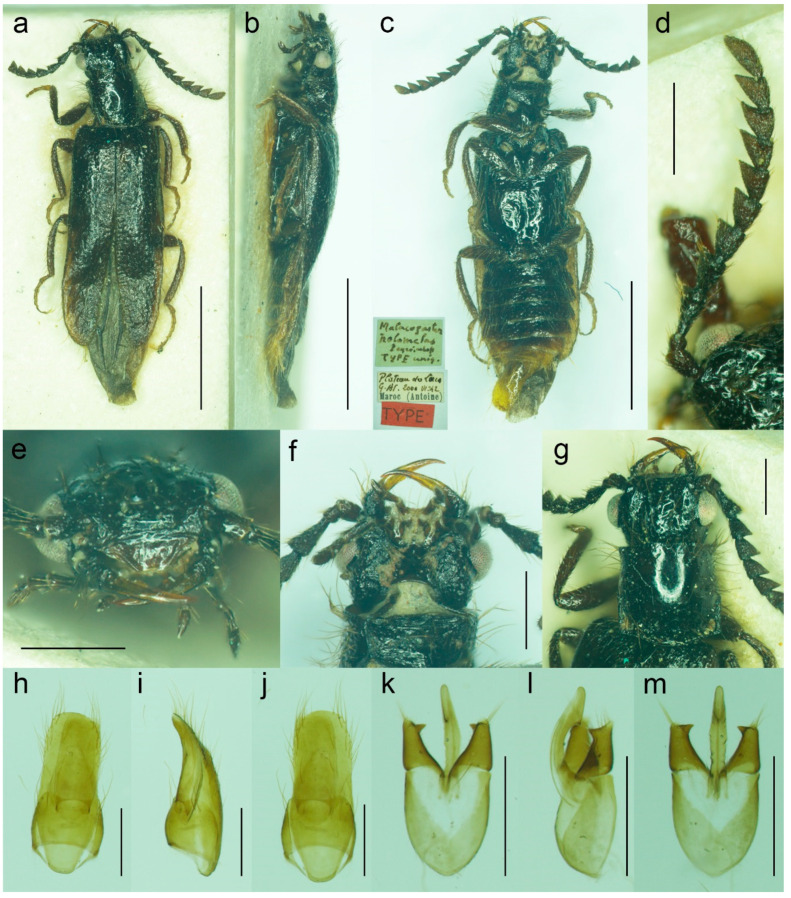
*Malacogaster holomelas* Peyerimhoff, 1949, male holotype. (**a**) Habitus, dorsal view; (**b**) habitus, lateral view; (**c**) habitus, ventral view; (**d**) right antenna; (**e**) head, frontal view; (**f**) head, ventral view; (**g**) pronotum, dorsal view; (**h**) genital capsule formed by tergites IX and X, and sternite IX, dorsal view; (**i**) genital capsule formed by tergites IX and X, and sternite IX, lateral view; (**j**) genital capsule formed by tergites IX and X, and sternite IX, ventral view; (**k**) aedeagus, dorsal view; (**l**) aedeagus, lateral view; (**m**) aedeagus, ventral view. Scale bars = (**a**–**c**) 2.0 mm; (**d**–**m**) 0.5 mm.

(Figure 8 and Figure 25a)

*Malacogaster holomelas* Peyerimhoff, 1949: 249/266 [32].

**Type material**. Holotype, male, “Plateau des Lacs/G. At. 2000 VI. 42/Maroc (Antoine)//Type [red printed label]//*Malacogaster*/*holomelas*/Peyerimhoff/Type uniq.” (MNHN).

**Type locality**. Morocco: Plateau des Lacs (near Imilchil).

**Other material examined**. We were able to study only the holotype of this easily recognizable species. Another specimen was reported by Kocher [34] from Moyen Atlas: Enjil (as “Engil”). The third known specimen was collected in Nzala on 10th April 2011 by H. Labrique, and it is deposited in the MHNL (identity confirmed based on the detailed photograph provided by H. Labrique).

**Differential diagnosis**. This is the only species of *Malacogaster* with a completely black pronotum (Figure 8g). All other species have a pronotum that is yellowish to reddish brown, with only some rare examples having a median portion of the pronotal disk somewhat darker (this is especially true for *M. bassii* from Algeria based on the description and drawing by Lucas [25]). Additionally, *M. holomelas* differs from all his congeners in having body more than 3.60 times as long as wide, and elytra combined more than 2.50 times as long as wide (all other species have body 2.40–3.20 times as long as wide, and elytra combined 1.55–2.25 times as long as wide).

**Diagnostic redescription**. Based on the holotype. Male. Body (Figure 8a–c) 5.10 mm long (non-type specimen in MNHL: 6.50 mm), 3.65 times as long as wide; dark brown to black, femora and tibiae slightly lighter, labrum and tarsi brown, apical segments of abdomen yellowish to reddish brown. Body pubescence yellowish brown to brown. Head 1.20 times as wide as anterior margin of pronotum, and 1.10 times as wide as pronotum measured at widest place. Fronto-clypeal region (Figure 8e,f) short and wide, apically widely concave; eyes relatively small, their minimum frontal separation 2.10 times maximum eye diameter; labrum large, subtrapezoidal, well visible, anteriorly slightly concave; antenna (Figure 8d) with antennomere III about 1.25 times longer than antennomere IV; median antennomeres 1.15–1.20 times as wide as long. Pronotum (Figure 8g) subquadrate, as wide as long when measured at widest place, narrowest at anterior third, widest subequally posteriorly and medially, with lateral sides bisinuate; elytra (Figure 8a) elongate, combined 2.55 times as long as wide, and 3.85 times as long as pronotal length. Abdominal sternite IX about 2.70 times as long as wide; tergite X elongate, 1.95 times as long as wide (Figure 8h–j). Aedeagus (Figure 8k–m) 2.25 times as long as wide; median lobe relatively slender, 1.10 times as long as phallobase, and 2.25 times as long as lateral portion of paramere; paramere relatively short, apically truncate, partly membranous, with latero-apical projection on inner side, apex slightly emarginate in lateral view; phallobase robust, relatively long, 0.55 times as long as whole aedeagal length, 1.25 times as long as wide, and 2.10 times as long as lateral portion of paramere.

**Variability**. The non-type specimen from Nzala (MHNL) has the pronotum relatively wider, about 1.10 times as wide as long when measured at the widest place.

**Distribution**. Morocco (Figure 25a).

**Literature**. Peyerimhoff (1949: 249/266): original description [32]; Pic (1951: 295): remarks [33]; Kocher (1956: 25): catalogue [34]; Bocak (2007: 210): catalogue [15]; Kundrata and Bocak (2019: 441): review [1].

#### 3.1.3. *Malacogaster maculiventris* Reitter, 1894

**Figure 9:**
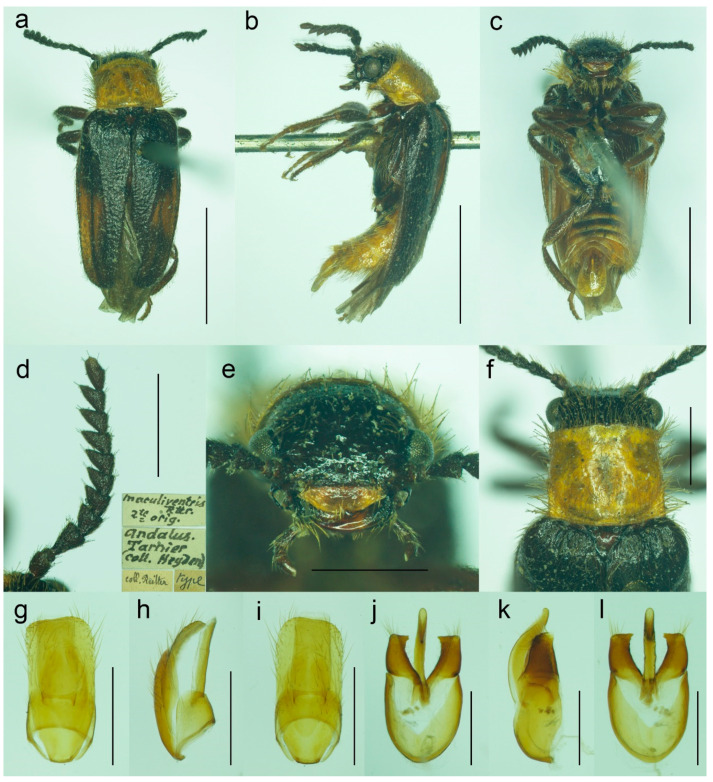
*Malacogaster maculiventris* Reitter, 1894, male lectotype. (**a**) Habitus, dorsal view; (**b**) habitus, lateral view; (**c**) habitus, ventral view; (**d**) right antenna; (**e**) head, frontal view; (**f**) pronotum, dorsal view; (**g**) genital capsule formed by tergites IX and X, and sternite IX, dorsal view; (**h**) genital capsule formed by tergites IX and X, and sternite IX, lateral view; (**i**) genital capsule formed by tergites IX and X, and sternite IX, ventral view; (**j**) aedeagus, dorsal view; (**k**) aedeagus, lateral view; (**l**) aedeagus, ventral view. Scale bars = (**a**–**c**) 3.0 mm; (**d**–**i**) 1.0 mm; (**j**–**l**) 0.5 mm.

**Figure 10:**
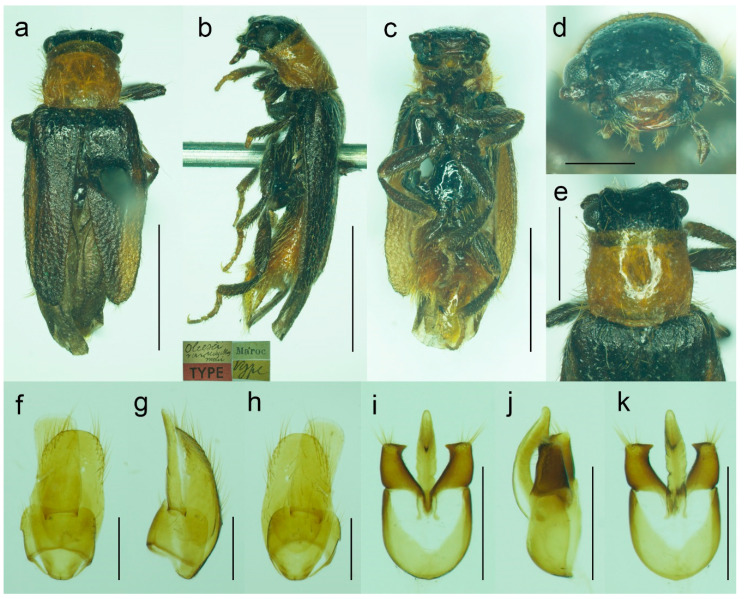
*Malacogaster maculiventris* Reitter, 1894. Male holotype of *Malacogaster olcesei* var. *reductus* Pic, 1949, syn. nov. (**a**) Habitus, dorsal view; (**b**) habitus, lateral view; (**c**) habitus, ventral view; (**d**) head, frontal view; (**e**) pronotum, dorsal view; (**f**) genital capsule formed by tergites IX and X, and sternite IX, dorsal view; (**g**) genital capsule formed by tergites IX and X, and sternite IX, lateral view; (**h**) genital capsule formed by tergites IX and X, and sternite IX, ventral view; (**i**) aedeagus, dorsal view; (**j**) aedeagus, lateral view; (**k**) aedeagus, ventral view. Scale bars = (**a**–**c**) 2.0 mm; (**d**, **f**–**k**) 0.5 mm; (**e**) 1.0 mm.

**Figure 11:**
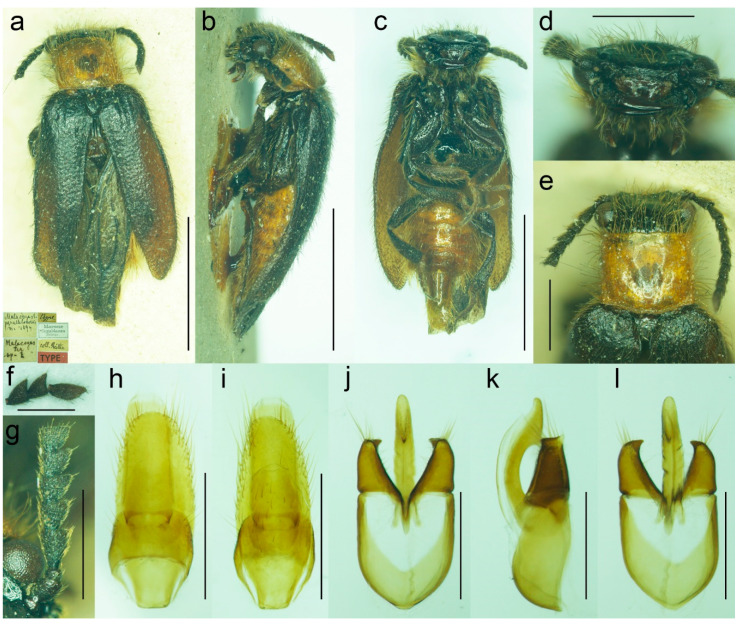
*Malacogaster maculiventris* Reitter, 1894. Male lectotype of *Malacogaster parallelocollis* Reitter, 1894, syn. nov. (**a**) Habitus, dorsal view; (**b**) habitus, lateral view; (**c**) habitus, ventral view; (**d**) head, frontal view; (**e**) pronotum, dorsal view; (**f**) apical antennomeres of left antenna; (**g**) basal and median antennomeres of left antenna; (**h**) genital capsule formed by tergites IX and X, and sternite IX, dorsal view; (**i**) genital capsule formed by tergites IX and X, and sternite IX, ventral view; (**j**) aedeagus, dorsal view; (**k**) aedeagus, lateral view; (**l**) aedeagus, ventral view. Scale bars = (**a**–**c**) 3.0 mm; (**d**,**e**,**g**–**i**) 1.0 mm; (**f**,**j**–**l**) 0.5 mm.

(Figure 9, Figure 10, Figure 11 and Figure 25b)

*Malacogaster maculiventris* Reitter, 1894: 4 [6].

*Malacogaster parallelocollis* Reitter, 1894: 4 [6], **syn. nov**.

*Malacogaster masculiventris*: Fuente, 1931: 64 [101] [unavailable name, incorrect subsequent spelling not in prevailing usage].

*Malacogaster olcesei* var. *reductus* Pic, 1951: 297 [33], **syn. nov**.

*Malacogaster reductus*: Löbl and Smetana, 2010: 25 [36].

**Type material**. *Malacogaster maculiventris*: Described based on an unknown number of specimens [6]. Lectotype by present designation, male, “*maculiventris*/Rttr./2 te = orig.//Andalus./Tarnier/(coll. Heyden)//coll. Reitter//type” (MNHN) (Figure 9). *Malacogaster olcesei* var. *reductus*: Holotype, male, “Maroc//type//*Olcesei*/v. *reductus*/mihi//TYPE [red printed label]” (MNHN) (Figure 10). *Malacogaster parallelocollis*: Described based on an unknown number of specimens [6]. Lectotype by present designation, male “Marocco/Casablanca/Reitter.//*Malacogas*/*ter*/sp.//coll. Reitter//type//*Malacogast*./*parallelocollis*/m. 1894//Type [red printed label]” (MNHN) (Figure 11).

**Type localities**. *Malacogaster maculiventris*: Spain: Andalusia. *Malacogaster olcesei* var. *reductus*: Morocco (without any further details). *Malacogaster parallelocollis*: Morocco: Casablanca.

**Other material examined**. **Spain**. One male, “Andalusien/Malaga/C. Ribbe.//coll. Leonhard//*Malacogas*-/*ter* spec?//*Malacogaster*/*maculiventris*/dat. Rich. Hicker Rtt.//1 Stück behalten” (SDEI); one male, “Algeciras/(Korb)//*Malacogast*./*maculiventr*.//coll. Leonhard//*Malacogast*./*maculiven*./*tris* Rtt.” (SDEI); one male, “Cordoba/Kraatz//*Malacogaster*/*maculiventris* Rtt./Det. Rich. Hicker” (SDEI); one male, “Churriana/nr airport Málaga/wasteland, 17. IV. 1983/E. I. S. UF 65” (PCRK); one male, “Ronda, Spain/G.C.C.//G. C. Champion/BMNH (E) 1927-409” (BMNH); one male, “Andalus//coll. Stierlin//*M. Passerinii*/Bassy//Ganglbauer/rev. 1908.//*Maculiventris*/Reitt.” (SDEI)”; one male, “Baena Cordoba/El Zambudio 30. 6. 2013/M. Baena leg.” (PCRK); one male, “Span. mer./Marbella/1-21/7 62” (MZLU); one male, “Algeciras (Cadiz), Hispania, J. de Ferrer leg. [underside: Getares, 1 Ago. 1986]” (MNCN); two males, “Algeciras (Cadiz), J. Aranaz P. leg. [underside: 11-6-79]” (MNCN). **Portugal**. One male, “Lusitania 1910/Faro VI./A. Schatzmayr/Coll. O. Leonhard” (SDEI); one male, “Vicinity of Almadena/W of Lagos/1/5/02, by sweeping ISM/02’ Alg. Port.//I. S. Menzies collection, BMNH (E) 2008-31//*Malacogaster maculiventris* det. M. Geiser 2016” (BMNH); one male, “Ericeira, Nr. Lisbon/Portugal/i. viii. 1970//BMNH (E)/1998-129/W R B Hynd” (BMNH); one male “Portugal/[further data unreadable]/Juni 2001//*Malacogaster*/*maculiventris* Reitt./det. G. Liberti XII. 2014” (MFNB); three males “Portugal/Algarve/Silves 1. 5. 1998/leg. O. Blochwitz//*Malacogaster*/*nigripes* Schaufuss/S. Kazantsev det. 2012” (MFNB); one male “P Algarve Fa/Portimao/02. 05. 2004/leg. K. Liebenow//*Malacogaster*/*nigripes* Schaufuss/S. Kazantsev det. 2012” (MFNB). **Gibraltar**. Nine males, “Gibraltar/J. J. Walker//G. C. Champion/BMNH (E) 1927-409” (BMNH); one male, “Gibraltar/J. J. Walker//G. C. Champion/BMNH (E) 1927-409//*Malacogaster* sp.//*Malacogaster maculiventris*/det. M. Geiser 2016” (BMNH); one male, “Gibraltar/J. J. Walker//G. C. Champion/BMNH (E) 1927-409//160” (BMNH); one male, “Gibraltar/J. J. Walker//G. C. Champion/BMNH (E) 1927-409//1334//*Malacogaster maculiventris*/det. M. Geiser 2016” (BMNH); two males, “Gibraltar/G. C. Champion collection/B.M. 1927-409” (one with the additional label: “*Malacogaster maculiventris*/det. M. Geiser 2016”) (BMNH). **Morocco**. One male, “Morocco—NE 10km/SSW Guercif, Moulouya/riv. 34°08′ N, 3°23′ W,/h = 380 m, 26. V. 2012,/Lg. A. Napolov & I. Roma//coll. A. Kopetz” (NKME); one male, “Morocco—NE 10km/SSW Guercif, Moulouya/riv. 34°08′ N, 3°23′ W,/h = 380 m, 27. V. 2012,/Lg. A. Napolov & I. Roma//coll. A. Kopetz” (NKME); one male, “Maroc 23. 5. 1995/Lareche env./S. Kadlec lgt.//ex coll. S. Kadlec/National Museum/Prague, Czech Republic” (NMPC); one male, “Morocco, Volubilis,/~500 m./20. V. 2002./leg. L. Nádai” (HNHM); one male, “13/7/44/P. Rotrou-Taza//*Malacogaster*/*Rotroui*. Kocher//Cotype [red label] [since we have not found the description of this species we believe it is a manuscript name]” (MNHN).

**Figure 12:**
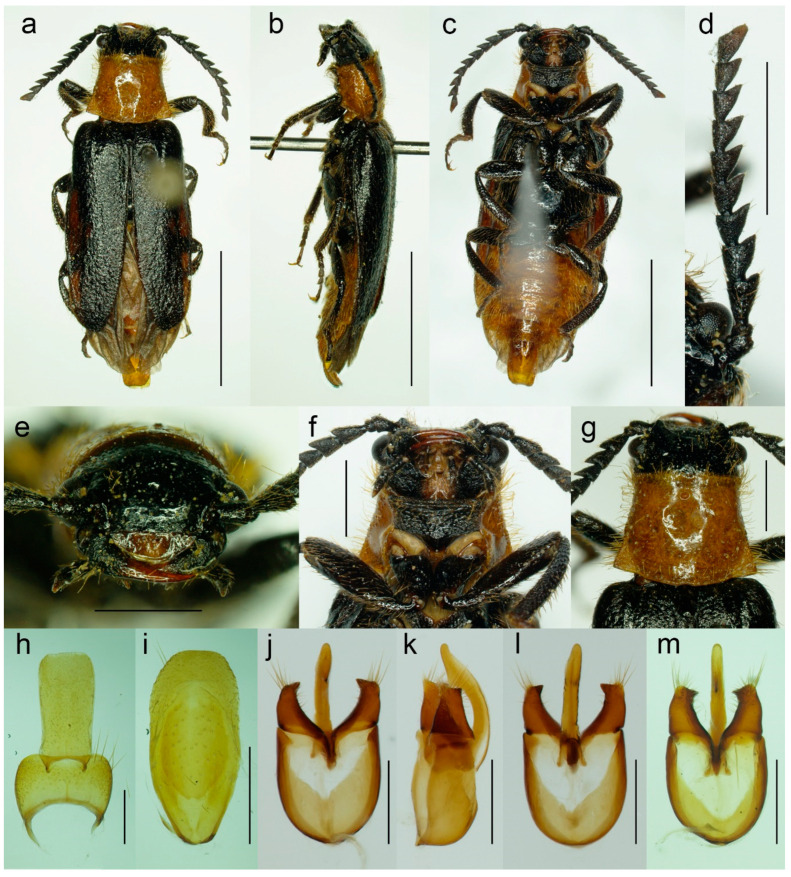
*Malacogaster nigripes* Schaufuss, 1867, male. Specimen from Altea, Spain. (**a**) Habitus, dorsal view; (**b**) habitus, lateral view; (**c**) habitus, ventral view; (**d**) left antenna; (**e**) head, frontal view; (**f**) head, ventral view; (**g**) pronotum, dorsal view. Specimen from Alcossebre, Spain. (**h**) abdominal tergites IX and X, dorsal view; (**i**) abdominal sternite IX, ventral view. Specimen from Altea, Spain. (**j**) Aedeagus, dorsal view; (**k**) aedeagus, lateral view; (**l**) aedeagus, ventral view. Specimen from Alcossebre, Spain. (**m**) Aedeagus, dorsal view. Scale bars = (**a**–**c**) 3.5 mm; (**d**) 1.5 mm; (**e**–**g**,**i**) 1.0 mm; (**h**,**j**–**m**) 0.5 mm.

**Figure 13:**
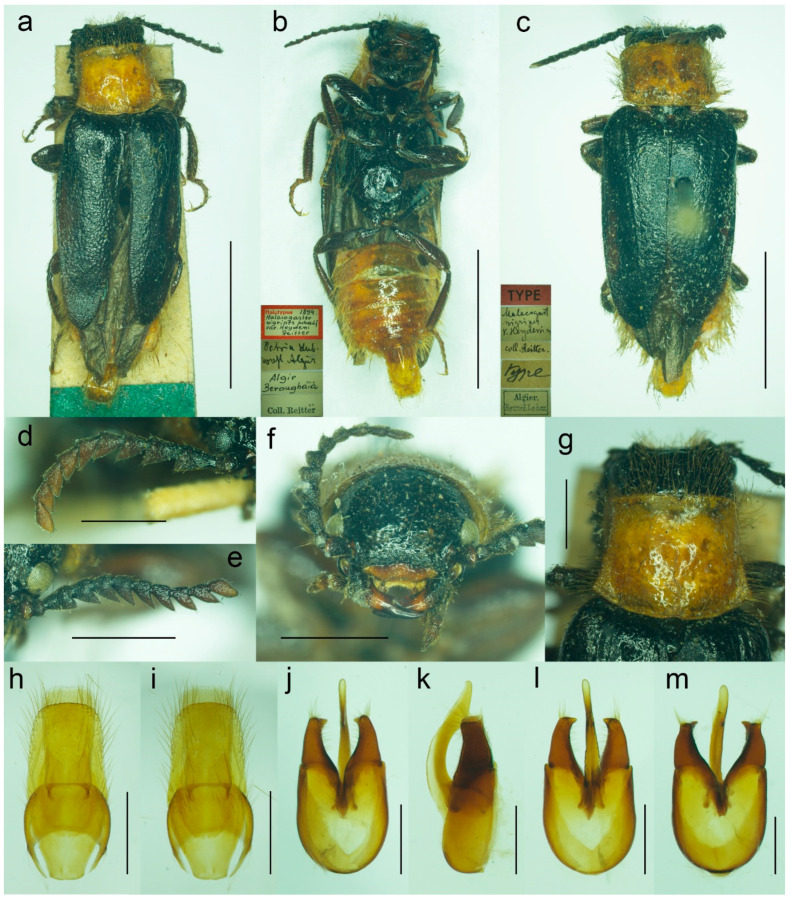
*Malacogaster nigripes* Schaufuss, 1867. Lectotype and paralectotype of of *Malacogaster nigripes* var. *heydeni* Reitter, 1894. (**a**) Lectotype habitus, dorsal view; (**b**) lectotype habitus, lateral view; (**c**) paralectotype habitus, dorsal view; (**d**) lectotype right antenna; (**e**) paralectotype left antenna; (**f**) paralectotype head, frontal view; (**g**) lectotype pronotum, dorsal view; (**h**) lectotype genital capsule formed by tergites IX and X, and sternite IX, dorsal view; (**i**) lectotype genital capsule formed by tergites IX and X, and sternite IX, ventral view; (**j**) lectotype aedeagus, dorsal view; (**k**) lectotype aedeagus, lateral view; (**l**) lectotype aedeagus, ventral view; (**m**) paralectotype aedeagus, dorsal view. Scale bars = (**a**–**c**) 4.0 mm; (**d**,**g**–**i**) 1.0 mm; (**e**,**f**) 1.5 mm; (**j**–**m**) 0.5 mm.

**Figure 14:**
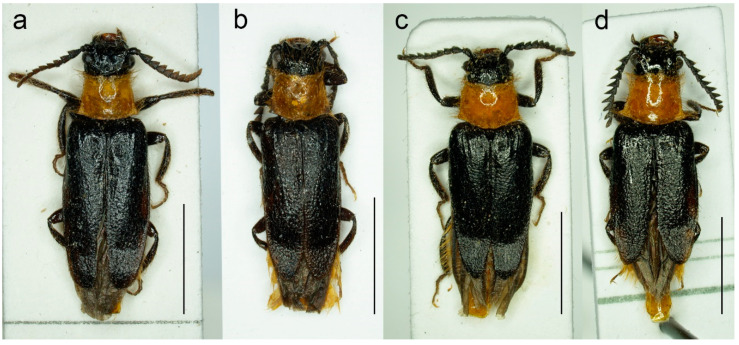
*Malacogaster nigripes* Schaufuss, 1867, male, habitus, dorsal view. (**a**) Specimen from Mallorca (PCRK); (**b**) Specimen from Mallorca (HNHM); (**c**) Specimen from Mallorca (NKME); (**d**) Specimen from Morocco (NKME). Scale bars = (**a**,**b**,**d**) 3.0 mm; (**c**) 3.5 mm.

**Figure 15:**
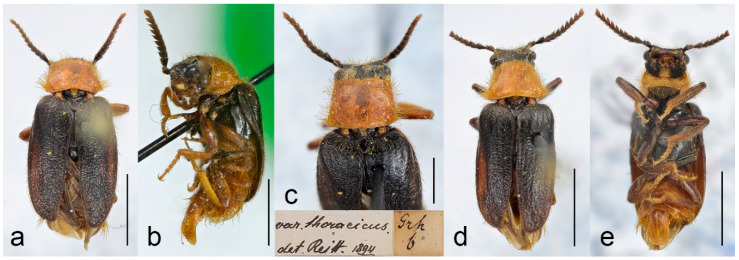
*Malacogaster passerinii* Bassi, 1834. Male lectotype of *Malacogaster passerinii* var. *thoracicus* Redtenbacher, 1858 (**a**) Habitus, dorsal view; (**b**) habitus, lateral view; (**c**) pronotum and basal portion of elytra, dorsal view. Specimen from Sicily (NHMW). (**d**) Habitus, dorsal view; (**e**) habitus, ventral view. Scale bars = (**a**,**b**,**d**,**e**) 3.0 mm; (**c**) 1.5 mm. Photographs courtesy of H. Schillhammer and M. Seidel (NHMW).

**Figure 16:**
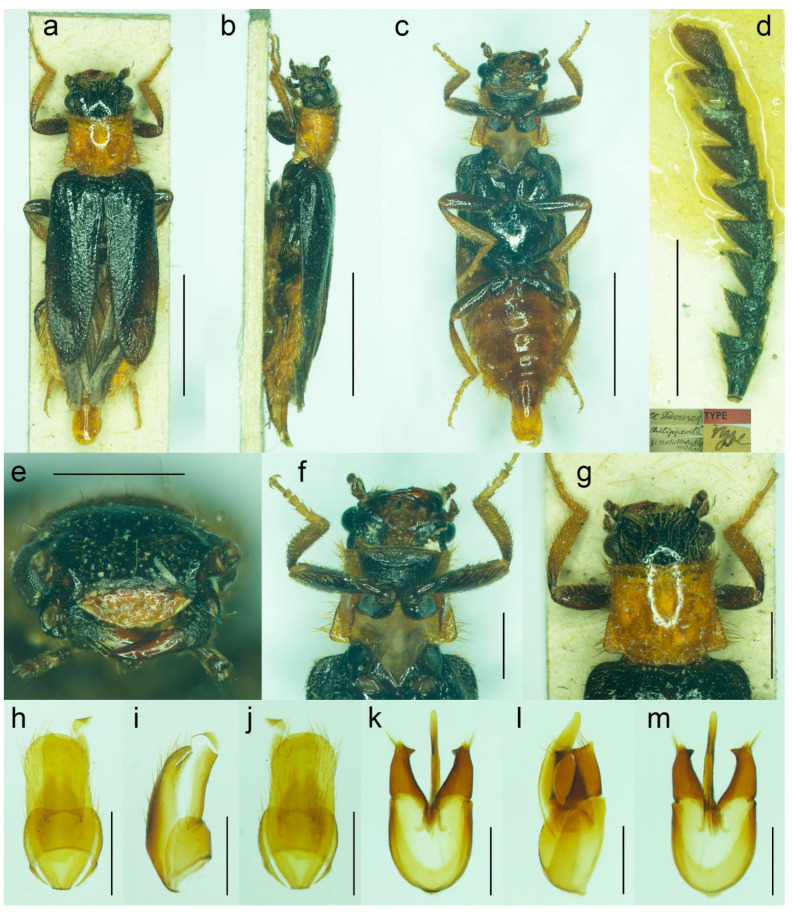
*Malacogaster passerinii* Bassi, 1834. Male holotype of *Malacogaster notativentris* Pic, 1949, syn. nov. (**a**) Habitus, dorsal view; (**b**) habitus, lateral view; (**c**) habitus, ventral view; (**d**) right antenna; (**e**) head, frontal view; (**f**) head and prothorax, ventral view; (**g**) pronotum, dorsal view; (**h**) genital capsule formed by tergites IX and X, and sternite IX, dorsal view; (**i**) genital capsule formed by tergites IX and X, and sternite IX, lateral view; (**j**) genital capsule formed by tergites IX and X, and sternite IX, ventral view; (**k**) aedeagus, dorsal view; (**l**) aedeagus, lateral view; (**m**) aedeagus, ventral view. Scale bars = (**a**–**c**) 3.0 mm; (**d**–**j**) 1.0 mm; (**k**–**m**) 0.5 mm.

**Figure 17:**
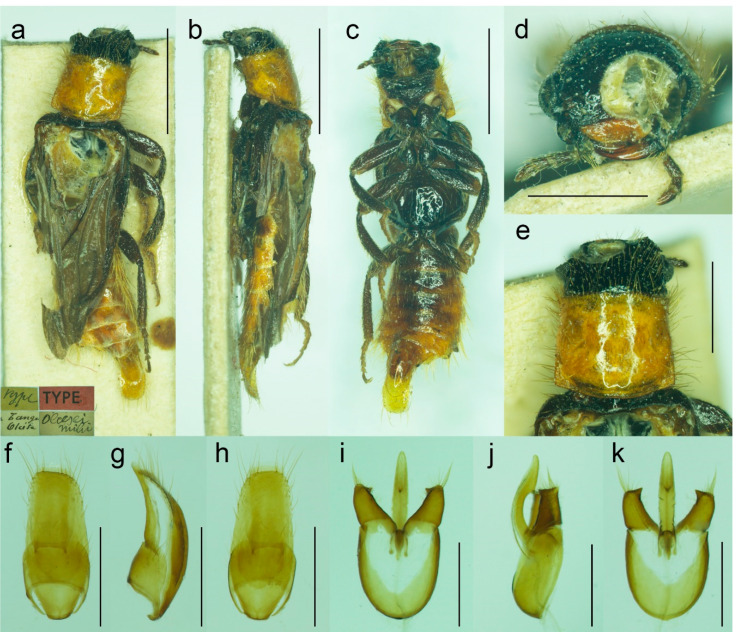
*Malacogaster passerinii* Bassi, 1834. Male lectotype of *Malacogaster olcesei* Pic, 1949, syn. nov. (**a**) Habitus, dorsal view; (**b**) habitus, lateral view; (**c**) habitus, ventral view; (**d**) head, frontal view; (**e**) pronotum, dorsal view; (**f**) genital capsule formed by tergites IX and X, and sternite IX, dorsal view; (**g**) genital capsule formed by tergites IX and X, and sternite IX, lateral view; (**h**) genital capsule formed by tergites IX and X, and sternite IX, ventral view; (**i**) aedeagus, dorsal view; (**j**) aedeagus, lateral view; (**k**) aedeagus, ventral view. Scale bars = (**a**–**c**) 2.0 mm; (**d**–**h**) 1.0 mm; (**i**–**k**) 0.5 mm.

**Figure 18:**
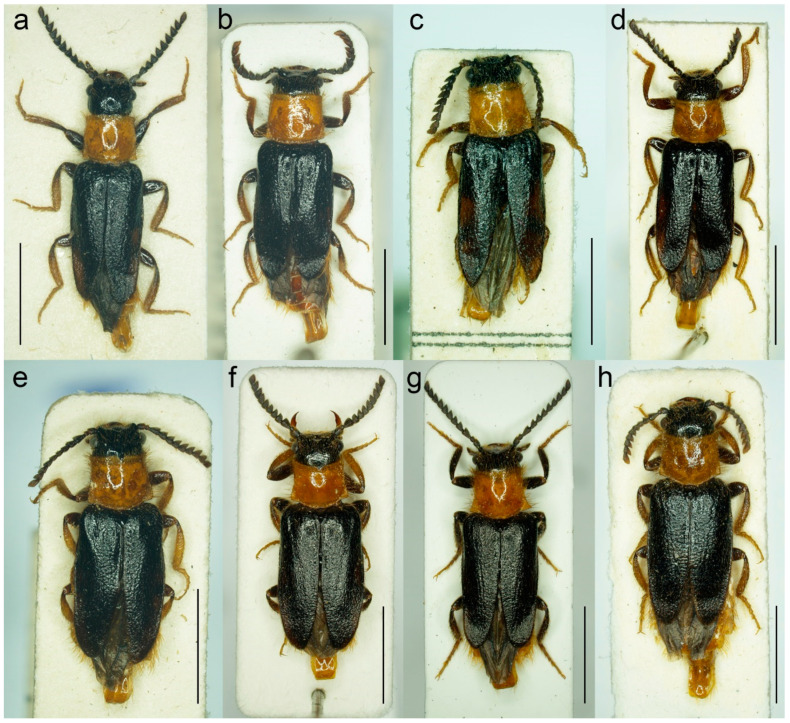
*Malacogaster passerinii* Bassi, 1834, male, habitus, dorsal view. (**a**) Specimen from Sardinia (MZLU); (**b**) specimen from Sardinia (HNHM); (**c**) specimen from Sicily (NMPC); (**d**) specimen from Sardinia (PCRK); (**e**) specimen from Tunisia (NMPC); (**f**) specimen from Tunisia (NMPC); (**g**) specimen from Tunisia (PCRK); (**h**) specimen from Spain (NMPC). Scale bars = (**a**) 2.5 mm; (**b**,**d**,**e**,**h**) 3.5 mm; (**c**) 3.0 mm; (**f**,**g**) 4.0 mm.

**Differential diagnosis**. This species differs from *M. nigripes* (Figure 12, Figure 13 and Figure 14) and *M. passerinii* (Figure 3, Figure 4, Figure 5, Figure 15, Figure 16, Figure 17 and Figure 18), which occur in the same area and have generally similar body size and coloration, in the subquadrate pronotum with subparallel sides (pronotum subtrapezoidal, usually distinctly narrower anteriorly and widest posteriorly in *M. nigripes* and *M. passerinii*), and the pronotum width at posterior angles 1.00–1.10 times width at anterior angles (1.15–1.30 times in *M. nigripes* and *M. passerinii*). Additionally, it differs from *M. nigripes* in yellowish brown to light brown elytral pubescence (reddish dark brown or dark brown to black in *M. nigripes*), and from the typical *M. passerinii* in more or less uniformly dark brown tibiae (tibiae darker basally and lighter apically in many *M. passerinii*) and relatively wider parameres at apex in dorsal view (parameres subapically distinctly narrowed in dorsal view in *M. passerinii*). Further, *M. maculiventris* has relatively larger eyes (often really distinctly surpassing sides of pronotum), with the minimal interocular distance 1.85–2.30 times maximum eye diameter (usually around 2.00–2.15 times; around 2.50 times in *M. nigripes* and *M. passerinii*). *Malacogaster theryi*, known only from the holotype from Morocco (Figure 22 and Figure 25a), shares the relatively larger eyes with *M. maculiventris* but differs in pronotum being distinctly widest posteriorly and with concave lateral sides.

**Diagnostic redescription**. Based on the lectotype. Male. Body (Figure 9a–c) 6.10 mm long, 2.75 times as long as wide; dark brown, legs brown, tarsi slightly lighter, labrum yellowish brown, abdomen yellowish to reddish brown, ventrites 1–5 medially dark brown. Body pubescence yellowish brown to brown. Head including eyes 1.05 times as wide as anterior margin of pronotum, and 0.95 times as wide as pronotum measured at widest place. Fronto-clypeal region (Figure 9e) short and wide, apically widely concave; eyes medium-sized, their minimum frontal separation 2.25 times maximum eye diameter; labrum large, subtrapezoidal, well visible, anteriorly slightly concave; antenna (Figure 9d) with antennomere III about 1.35 times longer than antennomere IV; median antennomeres about 1.40 times as wide as long. Pronotum (Figure 9f) subquadrate, 1.25 times as wide as long when measured at widest place, narrowest at one third after anterior angles, widest posteriorly, with lateral sides bisinuate; elytra (Figure 9a) relatively short, combined 1.90 times as long as wide, and 3.25 times as long as pronotal length. Abdominal sternite IX about 2.20 times as long as wide; tergite X elongate, twice as long as wide (Figure 9g–i). Aedeagus (Figure 9j–l) twice as long as wide; median lobe robust, 1.05 times as long as phallobase, and 2.40 times as long as lateral portion of paramere; paramere robust, truncate apically, with latero-apical projection on inner side; phallobase robust, 0.55 times as long as whole aedeagal length, 1.15 times as long as wide, and 2.20 times as long as lateral portion of paramere.

**Variability**. Body length of the examined specimens was 4.4–7.1 mm (holotype of *M. olcesei* var. *reductus* 4.4 mm, lectotype of *M. parallelocollis* 5.8 mm). Bahillo de la Puebla and López Colón [35] considered *M. maculiventris* a small species (4–5 mm long) but they also reported a specimen 6.9 mm long in the personal collection of R. Constantin (France). Here studied specimens are mostly around 5–6 mm but some are larger. Additionally, the species identity of the specimens reported by Bahillo de la Puebla and López Colón [35] should be re-evaluated based on the current concept of *M. maculiventris* (see below). Head including eyes is often distinctly wider than anterior portion of pronotum; 1.00–1.20 times as wide as anterior margin of pronotum, and 0.95–1.15 times as wide as pronotum measured at widest place. Median antennomeres are about 1.20–1.40 times as wide as long. The eyes vary from being medium-sized (lectotype of *M. maculiventris*, holotype of *M. olcesei* var. *reductus*) to relatively large; their minimum frontal separation is 1.85–2.30 times the maximum eye diameter. The pronotum is rather variable in shape; it is 1.00–1.25 times as wide as long when measured at the widest place, and although it is usually only slightly widest at the posterior angles, sometimes it is widest medially (holotype of *M. olcesei* var. *reductus* and lectotype of *M. parallelocollis*) or both medially and posteriorly (Figure 9f, Figure 10e and Figure 11e). The combined elytra are 1.70–2.05 times as long as wide. Aedeagus 1.85–2.20 times as long as wide, with a clear gradation from a relatively robust and short (e.g., the holotype of *M. olcesei* var. *reductus*) to a relatively narrow elongated shape of both aedeagus and phallobase (e.g., the lectotype of *M. parallelocollis*). The shape of paramere is also slightly variable; it is apically either obliquely straight or slightly concave in lateral view (Figure 9j–l, Figure 10i–k and Figure 11j–l). The abdominal ventrite 1 is usually dark but the ventrites 2–5 are either more or less uniformly lightly colored (as in the lectotype of *M. parallelocollis*) or to various extent medially dark. Especially older specimens have also some slightly darker spots on pronotum.

**Distribution**. Gibraltar, Portugal, Spain (Andalusia), Morocco (Figure 25b). This species was reported also from the Balearic Islands [35] but these records need confirmation.

**Literature**. Reitter (1894: 4): original description of *M. maculiventris* and *M. parallelocollis*, identification key [6]; Winkler (1925: 523): catalogue [also as *M. parallelocollis*] [14]; Fuente (1931: 64): catalogue, distribution [as *M. masculiventris*; sic!] [101]; Pic and Lindberg (1932: 3): catalogue [as *M. parallelocollis*] [102]; Pardo Alcaide (1945: 457): catalogue [31]; Cobos (1949: 568/580): distributional record, checklist [109]; Peyerimhoff (1949: 266): comparison with other species [also as *M. parallelocollis*] [32]; Pic (1951: 296/297): remarks, original description of *M. olcesei* var. *reductus* [also as *M. parallelocollis*] [33]; Kocher (1956: 24): catalogue [also as *M. olcesei* var. *reductus* and *M. parallelocollis*] [34]; Torres Sala (1962: 240): catalogue, distribution [113]; Kocher (1969: 43): catalogue [116]; Bahillo de la Puebla and López Colón (2005: 125): revision, identification key, distributional map, photographs of male habitus [35]; Bocak (2007: 210): catalogue [15]; Löbl and Smetana (2010: 25): catalogue [as *M. reductus*] [36]; Zapata de la Vega and Sánchez-Ruiz (2012: 125): catalogue, distributional maps [56]; Zapata de la Vega and Sánchez-Ruiz (2013: 180): catalogue [131]; Zapata de la Vega and Sánchez-Ruiz (2014: 157): catalogue [134]; Zapata de la Vega and Sánchez-Ruiz (2015: 186): catalogue [142]; Zapata de la Vega and Sánchez-Ruiz (2016: 197): catalogue [148]; Zapata de la Vega and Sánchez-Ruiz (2017: 275): catalogue [151]; Zapata de la Vega and Sánchez-Ruiz (2018: 31): catalogue [156]; Kundrata and Bocak (2019: 441): review [also as *M. parallelocollis*] [1]; Zapata de la Vega and Sánchez-Ruiz (2019: 74): catalogue [160]; Valcárcel and Prieto Piloña (2020: 317): remark [58].

**Remarks**. Earlier authors identified this species mainly based on several dark basal abdominal ventrites (especially medially). However, some other *Malacogaster* species show some degree of variability in the coloration of basal abdominal ventrites, and *M. maculiventris* is not an exception. For example, *M. passerinii*, which is a species with lightly colored, yellowish to light brown ventrites, has the basal ventrite (i.e., sternite II), which is usually not fully exposed, is dark brown (e.g., *M. passerinii*; Figure 5b), and in some specimens the dark color continues medially to sternite III or even further. Some other specimens of *M. passerinii* have even several basal ventrites distinctly dark brown (at least large median portions), similarly to the lectotype of *M. maculiventris*. Such specimens are not geographically bound to a single region; they can be found in Sardinia, Sicily, Tunisia, Algeria and Morocco. Similarly, the holotype of *M. theryi* (Figure 22c) as well as some *M. nigripes* have slightly darker abdominal ventrites. On the other hand, there are several specimens of *M. maculiventris* with more or less uniformly light coloration of abdomen, including the lectotype of *M. parallelocollis* (Figure 11c).

The current wide concept of *M. maculiventris* includes specimens from the southern part of the Iberian Peninsula and Morocco which have medium-sized to large eyes, more or less subparallel-sided pronotum, light coloration of setae on elytra, and dark tibiae. Most of these specimens also have dark basal abdominal ventrites. This concept therefore also includes specimens previously included in different species, i.e., *M. parallelocollis* and *M. olcesei* var. *reductus*. Although some characters such as the shape of pronotum, relative size of eyes, coloration of abdominal ventrites or the shape of aedeagus (mainly narrower versus relatively wider) are variable, they are mixed in the available material so that it is the best conclusion to treat all such specimens as a single species. It is unfortunate that the types of all three previously accepted species have slightly different pronota (Figure 9f, Figure 10e and Figure 11e) and it is understandable that without study of more (intermediate) specimens they were described as separate species. However, they just represent the examples of intraspecific variability which is relatively high in soft-bodied Drilini [155].

Although Pic [33] described *Malacogaster olcesei* var. *reductus* as a variety of *M. olcesei*, Löbl and Smetana [36] treated it as a separate species without any explanation. Here, we confirm that this taxon does not belong to *M. olcesei* (which we synonymize here with *M. passerinii*, see below) but rather to widely delimited *M. maculiventris*. Most apparent differences are the shape of pronotum (gradually widened posteriorly in *M. olcesei*, widest medially in *M. olcesei* var. *reductus*), the relative size of eyes (their minimal frontal interocular distance 2.65 times maximum eye diameter in *M. olcesei*, 2.25 times in *M. olcesei* var. *reductus*), and the shape of paramere (narrowed apically in dorsal view in *M. olcesei*, relatively wide apically in *M. olcesei* var. *reductus*) (Figure 10 and Figure 17).

The figure of pronotum of *Malacogaster* sp. from Oualidia, Morocco [48]; page 195, Figure 10, which was originally identified by Faucheux and colleagues as *M. passerinii* and later as *M. nigripes* (see [48] for more information), suggests that this species may in fact be *M. maculiventris*. However, we refrain here from making any conclusions until we can study the specimens and examine their relationships using a DNA-based approach.

It should be noted that *M. parallelocollis* was included neither in Wittmer’s catalogue of Drilidae [9] nor in Pic’s major work on *Malacogaster* [33].

#### 3.1.4. *Malacogaster nigripes* Schaufuss, 1867

(Figure 12, Figure 13, Figure 14 and Figure 25c)

*Malacogaster nigripes* Schaufuss, 1867: 85 [23].

*Malacogaster nigriceps*: Lucas, 1871: 23 [25] [unavailable name, incorrect subsequent spelling not in prevailing usage].

*Malacogaster nigripes* var. *heydeni* Reitter, 1894: 4 [6]. Synonymized with *M. nigripes* by Bocak (2007: 210) [15].

*Malacogaster nigripede*: Pardo Alcaide, 1945: 457 [31] [unavailable name, incorrect subsequent spelling not in prevailing usage].

*Malacogaster curticornis* Pic, 1951: 297 [33]. Synonymized with *M. nigripes* by Kocher (1956: 25) [34].

*Malacogaster longicornis* Pic, 1951: 297 [33]. Synonymized with *M. nigripes* by Kocher (1956: 25) [34].

**Type material**. *Malacogaster nigripes*: Holotype, male (collection unknown). *Malacogaster nigripes* var. *heydeni*: Lectotype by present designation, male, “Algir/Beroughaia//Patria Dub./[one word unreadable] Algier//Holotypus 1894./*Malacogaster*/*nigripes* Schauf./var. *Heydeni*/Reitter [printed label with red frame]//Coll. Reitter” (HNHM) (Figure 13a,b,d,g–l); paralectotype by present designation, male, “Algier./Reitter. Leder.//coll. Reitter.//type//*Malacogast*/*nigripes*/v. *Heydeni* m//Type [red printed label]” (MNHN) (Figure 13c,e,f,m). *Malacogaster curticornis*: Three syntypes, males (?MNHN). *Malacogaster longicornis*: Holotype, male (?MNHN). We have not been able to locate the type material of *M. nigripes*, *M. curticornis*, and *M. longicornis*.

**Type localities**. *Malacogaster nigripes*: Spain: Valencia. *Malacogaster nigripes* var. *heydeni*: Algeria: Berrouaghia (lectotype) [in the original description both Algeria: Berrouaghia and Morocco: As-Sawíra (as Mogador); we were not able to locate the specimen(s) from the second locality]. *Malacogaster curticornis*: Morocco: El Hajeb. *Malacogaster longicornis*: Morocco: Rabat.

**Other material examined**. **Spain (Iberian Peninsula)**. One male, “Hispania 6. 6. 91/Alcocéber [Alcossebre]/lgt. M. Krajčík//coll. general/National Museum/Prague, Czech Republic” (NMPC); one male, “Benidorm/20. IV./1982” (PCRK); one male, “Altea, 10 km N of/Benidorm/18. IV. 1982” (PCRK); one male, “Spanien//Ganglbauer/[further data unreadable] 1908//*M. nigripes*/Schauf.” (SDEI); one male, “XMOART, Melilla—Marruecos, F. Codina Padilla” [underside: V. 1956]//*Malacogaster nigripes* Schauf., F. Codina det., MNCN_Ent 167562” (MNCN); one male, “Navia de Suarna (Lugo)/23-VI-2004//*Malacogaster nigripes* Schaufuss, 1867/P. Bahillo Det. 2015//MNCN_Ent 132927” (MNCN); one male, “Provincia de Alicante/J. Lauffer” (MNCN); one male, “Provincia de Alicante/J. Lauffer//*Malacogaster nigripes*” (MNCN); one male, “Playa Campo Golf/Málaga II. VIII. 80/Bastazo et Vela leg.” (PCRK). **Spain (Mallorca)**. Two males, “I. Baleares, Mallorca/Puerto de Andraitz/23-31. V. 1972./leg. Dr. S. Mahunka//*Malacogaster*/*nigripes* SCHAUF./det. O. Merkl. 1991” (HNHM); one male, “Capdella ca 15 km/W. of Palma/9/13-VI-1975” (PCRK); one male, “E BAL Mallorca/Can Picafort/05. 05. 2012/leg. K. Liebenow” (NKME); one male, “ex coll./Dr. Kallert/Hamburg//Spanien/Balearen./Mallorca.” (NMPC); two males, “*M. nigripes* Schauf./Mallorca” (MNCN); one male, “Mallorca//*Malacogaster nigripes* Schauf. (vide Pic)” (MNCN); one male, “Palma, V-1908, Lozano” (MNCN). **Morocco**. One male, “Morocco, Moyen Atlas/Mt.r., 57 km SW Guercif,/33°49’ N,3°43’ W,/h = 1310 m, 29. V. 2012,/Lg. A. Napolov & I. Roma//coll. A. Kopetz” (NKME); one male (examined from the photograph), “Maroc oriental—Beni/Snassen occidentaux, rte/de Dar Moussa en venant/d’Aklim; stat° 16b/34°48′ N–02°39′ W/230 m; 25.03.2007//Mission 2007/H. Labrique et/G. Chavanon” (MHNL). **Algeria**. One male, “Algérie/Lambèze/L. Bleuse/Juin 1885” (PCRK); one male “Algeria/Ham. Meser//*Malacogaster*/*nigripes*/Frm. d. Schauf.” (MFNB); one male “Algier/Quedenfeldt//Algir/[further data unreadable]//*M. nigripes*” (MFNB); one male, “ex coll./Dr. Kallert/Hamburg//nordwestliches/Mittel Algerien/Hammam Rhi/ra.” (NMPC).

**Differential diagnosis**. This species shares with *M. passerinii* (Figure 3, Figure 4, Figure 5 and Figure 18) the overall body coloration, body shape, and the pronotum which is gradually widened posteriorly, the relative size of eyes, and paramere subapically narrowed in dorsal view and apically truncate in lateral view. They differ in the coloration of elytral pubescence (especially from basal third to apex) which is dark reddish brown to black in *M. nigripes* and yellowish to reddish brown in *M. passerinii* (but see Remarks). Additionally, *M. nigripes* has tibiae always uniformly dark brown to black while *M. passerinii* often has apical half of tibia lighter, although this character is not universally valid. *Malacogaster nigripes* differs from another relatively common and generally similar species, *M. maculiventris*, in having relatively smaller eyes, with the minimal interocular distance 2.40–2.95 times the maximum eye diameter (1.85–2.30 times in *M. maculiventris*), the pronotum subtrapezoidal, usually distinctly narrower anteriorly and widest posteriorly (subquadrate pronotum with subparallel to slightly rounded sides in *M. maculiventris*), the pronotum width at posterior angles 1.15–1.30 times width at anterior angles (1.00–1.10 times in *M. maculiventris*), darker elytral pubescence, and parameres subapically distinctly narrowed in dorsal view (relatively wider parameres at apex in a dorsal view in *M. maculiventris*) (Figure 9). *Malacogaster theyri* from Morocco has the similar shape of pronotum but differs from *M. nigripes* in having large eyes, with their minimal frontal separation 1.85 times maximum eye diameter (Figure 22).

**Diagnostic redescription**. Based on the material listed above. Male. Body (Figure 12a–c, Figure 13a–c and Figure 14a–d) 4.60–8.80 mm long, 2.55–2.95 times as long as wide; antennae brown to dark brown, scape and pedicel dark brown, head dark brown to black, pronotal disk and hypomeron yellowish to reddish brown, scutellum, elytra and thorax underside dark brown to black, legs with coxae mostly dark brown, only apically light brown, femora dark brown, tibiae brown to dark brown, tarsi brown to reddish brown, abdominal ventrites yellowish to reddish brown. Body pubescence long, yellowish, only elytral pubescence reddish dark brown to black, sometimes lighter basally. Head 1.00–1.10 times as wide as anterior margin of pronotum, and 0.85–0.95 times as wide as pronotum measured at widest place. Fronto-clypeal region (Figure 12e,f and Figure 13f) short and wide, apically widely concave; eyes relatively small, their minimum frontal separation about 2.40–2.95 times maximum eye diameter; labrum large, subtrapezoidal, well visible, anteriorly slightly concave; antenna (Figure 12d and Figure 13d,e) with antennomere III about 1.20–1.35 times longer than antennomere IV; median antennomeres 1.20–1.50 times as wide as long. Pronotum (Figure 12g and Figure 13g) more or less subtrapezoidal, 1.20–1.35 times as wide as long when measured at widest place, narrowest at one third after anterior angles, widest posteriorly, with lateral sides usually slightly bisinuate; elytra (Figure 12a and Figure 13a,c) elongate, combined 1.65–2.05 times as long as wide, and 2.95–3.50 times as long as pronotal length. Abdominal sternite IX 2.15–2.20 times as long as wide; tergite X 1.85–1.90 as long as wide (Figure 12h,i and Figure 13h,i). Aedeagus (Figure 12j–m and Figure 13j–m) 2.00–2.15 as long as wide; median lobe 1.20–1.25 times as long as phallobase, and 2.50–2.70 times as long as lateral portion of paramere; paramere robust, with distinct latero-apical projection on inner side, subapically narrowed in dorsal view, truncate apically, apex either almost straight or slightly concave; phallobase robust, approximately 0.55 times as long as whole aedeagal length, 1.10–1.20 times as long as wide, and 2.15–2.25 times as long as lateral portion of paramere.

**Variability**. This species is 4.60–8.80 mm long, with larger specimens known mainly from Algeria (former var. *heydeni*). Eyes are relatively small, their usual minimum frontal separation is about 2.50 times maximum eye diameter; however, it can be slightly less but also considerably more, especially in the larger specimens from Algeria (up to 2.90 times). The shape of the pronotum is slightly variable, and it is 1.20–1.35 times as wide as long when measured at the widest place, and also more or less subtrapezoidal, with width at posterior angles 1.15–1.30 times width at anterior angles. The elytral pubescence varies from reddish dark brown through dark brown to black.

**Distribution**. Spain (Iberian Peninsula, Mallorca), Algeria, Morocco (Figure 25c). Zanon [7] reported this species from the Cyrenaica region of Libya (mentioned also by Faucheux et al. [146]); however, this record probably belongs to *M. ruficollis* which has also dark legs and is known only from that area. This species has been recorded from Italy (first from Sicily and later also from Sardinia, see e.g., [124,164]) but we believe that it is based on the specimens of *M. passerinii* with darker legs which also occur in the northern coast of Africa. However, the *passerinii*/*nigripes* complex urgently needs detailed investigation (see Remarks).

**Literature**. Schaufuss (1867: 85): original description of *M. nigripes* [23]; Gemminger (1869: 1684): catalogue [77]; Lucas (1871: 23): comparison with other species [also as *M. nigriceps*; sic!] [25]; Marseul (1877: 42): catalogue [81]; Moragues (1889: 24): catalogue [85]; Reitter (1894: 4): identification key, original description of *M. nigripes* var. *heydeni* [6]; Medina (1895: 44): catalogue [87]; Olivier (1910: 4): catalogue [8]; Zanon (1922: 123): catalogue [7]; Dodero (1925: 7): comparison with other species [29]; Winkler (1925: 523): catalogue [also as *M. nigripes* var. *heydeni*] [14]; Cros (1930: 133): comparison with other species, taxonomic remark [also as *M. passerinii* var. *nigripes*] [4]; Gridelli (1930: 97): catalogue, remark [100]; Fuente (1931: 64): catalogue, distribution [101]; Wittmer (1944: 204): catalogue [9]; Pardo Alcaide (1945: 457): catalogue, comparison with other species, drawing of male genitalia [also as *M. nigripede*; sic!] [31]; Pic (1951: 295): remarks, comparison with other species [33]; Kocher (1956: 25): catalogue [also as *M. nigripes* var. *heydeni*] [34]; Gridelli (1960: 386): catalogue [112]; Lo Valvo and Massa (1995: 883): checklist [120]; Poggi (1995: 6): checklist [121]; Sparacio (1997: 66): catalogue, remark [122]; Poggi (2003: online): catalogue [124]; Bahillo de la Puebla and López Colón (2005: 125): revision, identification key, distributional map, photographs of male habitus and antenna [35]; Bocak (2007: 210): catalogue, *M. nigripes* var. *heydeni* as a synonym [15]; Faucheux and Agnas (2008: 109): hypermetamorphosis, larval and female descriptions, distributional map, drawing of larval cerci, photographs of larvae and females [38]; Faucheux and Agnas (2011: 79): biology of larvae and females, photographs of female mouthparts [39]; Zapata de la Vega and Sánchez-Ruiz (2012: 125): catalogue, distributional maps, *M. nigripes* var. *heydeni* as a synonym [56]; Zapata de la Vega and Sánchez-Ruiz (2013: 180): catalogue [131]; Faucheux and Agnas (2014: 258): remark [132]; Faucheux and Kundrata (2014: 97): morphology of female antenna, photograph and drawing of female antenna [133]; Zapata de la Vega and Sánchez-Ruiz (2014: 157): catalogue [134]; Faucheux (2015: 188): remark [137]; Zapata de la Vega and Sánchez-Ruiz (2015: 186): catalogue [142]; Agnas and Faucheux (2016: 180): biology, reproduction, photographs of egg-laying female [40]; Faucheux (2016: 165): antennal glands, photographs of perforated plates on the male antenna [41]; Faucheux (2016: 201): hypermetamorphosis, identification problem, photographs of female immature stages, female habitus, female hypermetamorphosis [42]; Faucheux (2016: 221): biology and ecology [43]; Faucheux (2016: 229): antennal morphology, photographs of male and female anntennal morphology [44]; Faucheux (2016: 267): larval remark, identification problem [144]; Faucheux (2016: 288): female antennal morphology, identification problem, photographs and drawings of female head and antenna [45]; Faucheux (2016: 311): male antennal morphology, comparison with other species, identification problem, photographs and drawings of male habitus, head, and antenna [46]; Faucheux and Agnas (2016: 60): hypermetamorphosis, photographs of male and female immature stages [47]; Faucheux and Ballardini (2016: 187): biology, mating, breeding, identification problem, photographs of male, female, and larval habitus, copulation, male head, mouthparts, pronotum, elytra, abdomen, and genitalia [48]; Faucheux and Beaulieu (2016: 102): hypermetamorphosis, photographs of male and female hypermetamorphosis, and larvae [49]; Faucheux and Beaulieu (2016: 107): primary larva antennae and associated sensilla, photographs and/or drawings of primary larva head and antenna [50]; Faucheux and Beaulieu (2016: 149): antennal sensilla of adult male, photographs of male head, pronotum, and antenna [145]; Faucheaux et al. (2016: 121): mouthparts and associated sensilla of primary larva, photographs of primary larva habitus, head, antenna, and mouthparts [51]; Faucheaux et al. (2016: 1/54): larval and adult morphology, ecology, distributional map, comparison with other species, photographs of immature stages, and male and female habitus [also as *M. passerinii nigripes*] [146]; Zapata de la Vega and Sánchez-Ruiz (2016: 178): catalogue [148]; Faucheux (2017: 1): female mouthparts and sensilla, biology, taxonomic remark, photographs and drawings of female head and mouthparts [52]; Faucheux (2017: 1): male mouthparts and sensilla, biology, taxonomic remark, photographs and drawings of male head and mouthparts [53]; Faucheux (2017: 1): female mouthparts, comparison with other species, taxonomic remark [150]; Zapata de la Vega and Sánchez-Ruiz (2017: 275): catalogue [151]; Faucheux (2018: 1): antennal morphology of female immature stages, remark on biology, taxonomic remark, photographs and drawings of antennal morphology in female immature stages and adult [54]; Faucheux and Agnas (2018: 1): teratology of female antennae, taxonomic remark, photographs and drawings of abnormal female antennae [55]; Zapata de la Vega and Sánchez-Ruiz (2018: 31): catalogue [156]; Kundrata and Bocak (2019: 441): review [1]; Zapata de la Vega and Sánchez-Ruiz (2019: 74): catalogue [160]; Chavanon (2020: 69): catalogue [161]; Valcárcel and Prieto Piloña (2020: 317): distributional remark, distributional map, photograph of male habitus, remark on *M. nigripes* var. *heydeni* [58]; Dal Cortivo et al. (2021: 69): key [163]; Poggi (2021: online): catalogue [164].

**Remarks**. Schaufuss [23] described *M. nigripes* and compared it with similar *M. passerinii*. He wrote these two species differ in the shape of pronotum (anteriorly narrowed in *M. nigripes*) and the coloration of the tibiae. In his key to the *Malacogaster* species, Reitter [6] separated both species based on the coloration of legs, especially tibiae, and elytral pubescence; and defined *M. passerinii* also as having anteriorly narrowed pronotum. Cros [4] proposed that *M. nigripes* could be conspecific with *M. passerinii* which, according to him, differs mainly in the coloration of pronotum, legs and elytral pubescence. Even in later cases when *M. nigripes* and *M. passerinii* were considered separate species, they were always distinguished from each other based on the body and pubescence coloration (e.g., [35]). We found minimal differences in the body coloration of these two species; although the typical *M. nigripes* are generally somewhat darker, this is absolutely not a universal character. Specimens of *M. nigripes* have tibiae always uniformly dark brown to black while typical *M. passerinii* have apical half of tibia more or less distinctly lighter, although many specimens, especially from northern Africa, have tibiae also uniformly dark brown. Regarding the elytral pubescence (especially from the basal third to apex of elytra), it is dark reddish brown to black in *M. nigripes*, and usually yellowish to reddish brown in *M. passerinii*. However, the differences in coloration of elytral pubescence are rather subtle in some specimens of *M. nigripes* and *M. passerinii* from Mallorca. Further, there are some specimens from the same locality which differ slightly in the coloration of elytral pubescence (e.g., two specimens from El Kantara, Algeria) and there are a few specimens from Algeria with darker coloration of legs and reddish brown elytral pubescence which are not easy to reliably assign to *M. nigripes* or *M. passerinii*. We tentatively keep such specimens in *M. passerinii* until the taxonomic situation of the *passerinii*/*nigripes* complex is solved using more specimens and the DNA-based approach in future. Both species share the shape of body, pronotum which is narrowed anteriorly and usually gradually widened posteriorly, relatively small to medium-sized eyes, and paramere subapically narrowed in dorsal view and apically truncate in lateral view. Although the paramere is not so distinctly narrowed apically in most examined specimens of *M. nigripes*, the differences are really very subtle; moreover, this character is not universal. Despite all this, we prefer to keep both species as valid until the detailed study focused on these widely distributed species is carried out.

Specimens from Oualidia, Morocco, which were identified as *M. nigripes* by Faucheux and Ballardini [48] (and earlier as *M. passerinii*; see [48]), might in fact belong to *M. maculiventris* (see Remarks under the latter species for more information).

Reitter [6] described a variety of *M. nigripes* from Algeria and named it *heydeni*. Surprisingly, there is no remark about *heydeni* in Wittmer`s Catalogue of Drilidae [9]. Bocak [15] listed *heydeni* as a synonym of *M. nigripes*. Both type specimens and the specimens identified earlier as var. *heydeni* have darker body coloration and also dark elytral pubescence, have very small eyes, with the minimal interocular distance 2.80–2.95 times maximum eye diameter, and the paramere with moderately narrowed apices. We therefore keep *heydeni* as a synonym of *M. nigripes*. There is a variability in the shape of pronotum among the studied specimens, with some of them having the pronotum less apparently widened posteriorly. When selecting the lectotype of *M. nigripes* var. *heydeni*, we followed the Recommendation 74E of the Code [60], which, among others, says that a syntype of known locality should be preferred to one of unknown origin (Algeria: Berrouaghia vs. Algeria only).

Pic [33] reported three males from El Hajeb, Morocco which were of different body size (5–8 mm) and had more or less straight thorax and short and thick antennae, and a single male from Rabat, Morocco, which had long antennae. He wrote that he did not want to describe them as new species; however, he proposed names for them (*M. curticornis* and *M. longicornis*, respectively) in case it appears later that they are indeed new species. Kocher [34] synonymized them both with *M. nigripes*. Since we have not been able to find the type specimens of *M. curticornis* and *M. longicornis*, we tentatively keep them as synonyms of *M. nigripes* as proposed by Kocher [34] although they may in fact belong to other species.

#### 3.1.5. *Malacogaster passerinii* Bassi, 1834

(Figure 1, Figure 2, Figure 3, Figure 4, Figure 5, Figure 15, Figure 16, Figure 17, Figure 18 and Figure 25d)

*Ctenidion ruficollis*: Dejean, 1833: 104 [21] [unavailable name, published without description, attributed to Hoffmansegg]. See e.g., Gemminger (1869: 1684) [77].

*Ctenidion thoracicum* [sic!]: Dejean, 1833: 104 [21] [unavailable name, published without description]. See e.g., Bassi (1834: pl. 99) [18].

*Malacogaster passerinii* Bassi, 1834: pl. 99 [18].

*Malacogaster thoracica* Redtenbacher, 1858: 525 [20].

*Melacogaster* (sic!) *thoracicus* [sic!]: Schaufuss, 1867: 86 [23] [erroneously attributed to Rossi].

*Malacogaster passerinii* var. *thoracica*: Gemminger, 1869: 1684 [77].

*Malacogaster passerini*: Reitter, 1894: 4 [6] [unavailable name, incorrect subsequent spelling not in prevailing usage].

*Malacogaster passerinii* var. *thoracicus* [sic!]: Olivier, 1910: 4 [8].

*Malacogaster pesserinii*: Cros, 1925: 302 [30] [unavailable name, incorrect subsequent spelling not in prevailing usage].

*Malacogaster notativentris* Pic, 1951: 296/297, **syn. nov**. [33]

*Malacogaster olcesei* Pic, 1951: 296, **syn. nov**. [33]

*Malacogaster thoracinus* [sic!]: Zapata de la Vega and Sánchez-Ruiz, 2012: 125 [56] [unavailable name, incorrect subsequent spelling not in prevailing usage].

**Type material**. *Malacogaster passerinii*: Holotype, male (collection unknown). *Malacogaster thoracica*: Described based on an unspecified number of specimens, males. At least one specimen located at NMHW (examined only based on the photographs kindly provided by the curators of NHMW; Figure 15). Lectotype by present designation, male, “Grh/b//var. *thoracicus*./det. Reitt. 1894” (NHMW). *Malacogaster notativentris*: Holotype, male, “Philippeville [Skikda]//ex Favarcq.//type//v. *notativentris*/mihi//Type [red printed label]” (MNHN) (Figure 16). *Malacogaster olcesei*: Described based on a half dozen specimens. Only a single specimen available for our study (Figure 17). Lectotype by present designation, male, “Tanger/Olcése//type//*Olcesei*/mihi//Type [red printed label]” (MNHN).

**Type localities**. *Malacogaster passerinii*: Italy: Sicily, Trapani. *Malacogaster thoracica*: Italy: Sicily. *Malacogaster notativentris*: Algeria: Skikda. *Malacogaster olcesei*: Morocco: Tangier.

**Other material examined**. **Italy (Sicily)**. Two males, “Palermo/10. 3. 918.//*Malacog*./*Passerinii*” (NMPC); one male, “Mettina [Messina]//coll. Hlisnikovský/P5/720/49” (NMPC); one male, “12304/*Malacogaster*/*Passerinii*/[further data unreadable] Bassi Sicl.” (NMPC); one male, “Sicilia//coll. Hlisnikovský/P5/720/49” (NMPC); one male, “*Malacog*./*Passerini* [sic!]//Sicilia” (NMPC); one male, “E. Merkl/Neu Bogsán//Sicilien//*Malacogaster*/v. *thoracicus* [sic!] R.” (NMPC); one male, “Sicilia/M. [further data unreadable]/E. Ragusa 5//Sammlung/Dr. J. B. Jörger/Masans-Chur/1957” (NHMB); one male, “*Passerinii*/Sicil. Bassi.” (NMPC); one male, “Sici-/lia//*Malacogaster*/*Passerini* [sic!]” (NMPC); one male, “Bowring 63.47*//*Malac. passerinii* Sicily” (BMNH); one male, “Sicilia//ex Mus. Murray//Fry coll. 1905/100” (BMNH); one male, “Bowring 63.47*//Europe//*Malacogaster passerinii* Bassi” (BMNH). Three specimens (var. *thoracicus* [sic!]) examined only based on the photographs kindly provided by the curators of NHMW: one male, “Sartorius/1876//var. *thoracicus*. [sic!]/det. Reitt. 1894” (NHMW); one male, “Dup./l. 280//var. *thoracicus*. [sic!]/det. Reitt. 1894” (NHMW); one male, “Mann/1858/Sicilia//var. *thoracicus*. [sic!]/det. Reitt. 1894” (NHMW). one male “Sicilien//*Malacogaster*/*Passerinii*/Bassi” (MFNB); one male “Sicilien/Agrigent/29. Mai 1939” (MFNB); one male, “IT. Sicilie NW/Citta del Mare/11.–18.5.13/Ing. Brokeš lgt.” (PCAL). **Italy (Sardinia)**. Two males, “U. Lostia/Sardinia//*Passerinii*/Rossi/Coll. Reitter” (HNHM); one male, “WW—Sardinien/Alghero//31. V. 1982/H. J. Bremer leg.//*Malacogaster*/*passerinii* Bassi/det. O. Merkl. 1990” (HNHM); three males, “Sardinien/S. Glorgio. Palm/19-27. 6. 1967” (MZLU); two males, “Sardinien/S. Glorgio. Palm/19-27. 6. 1967” (MZLU); two males, “Sardinien/Alghero. Palm/15-28. 6. 1967” (MZLU); one male, one female, “Damry.//Sassari 15-VI-00/mûrs caserne/étaient accouplis” (MNHN). **Italy (exact part not specified)**. Two males, “Italia/coll. E. Friv.//*Malacogaster*/*Passerinii* Bassi/coll. E. Frivaldszky//FRIV./4074” (HNHM). **Algeria**. One male, “Algier//coll./Dr. J. Fodor//*Malacogaster*/*passerini* [sic!]/det. Wittmer//*Malacogaster*/*Passerini* [sic!]//Drilidae” (HNHM); one male, “Bône//v. *thoracicus* [sic!]/Redt./Coll. Reitter” (HNHM); one male, “Algerien/Bône/10. 5. 06/W. Liebmann//Coll./W. Liebmann/Arnstadt” (SDEI); one male, “El Guerrah/Algeria, G.C.C.//G. C. Champion/BMNH (E) 1927-409” (BMNH); one male, “Bome [sic!]//v. *thoracicus* [sic!]/Redt./Coll. Reitter” (HNHM); one male, “Algeria 4.5/Sidi Ferdj/A. Olexa 1987//ex coll. R. Dunda/National Museum/Prague, Czech Republic” (NMPC); one male, “Hadjar/IV 00//Algier//coll. Stierlin//Ganglbauer/rev. 1908.//*nigripes*/Schauf.” (SDEI); one male, “Hadjar/IV 00//coll. Stierlin//Ganglbauer/rev. 1908.” (SDEI); one male, “Hadjar/IV 00//coll. Stierlin//Ganglbauer/rev. 1908.” (SDEI); one male, “Bone/[further data unreadable]//443//Coll. Kraatz” (SDEI); one male, “Bône/Dr. W. Horn/12696.//Coll. Kraatz//= *Malacogaster*/*nigripes* Schf.” (SDEI). **Tunisia**. One male, “3 km E. Tabarka/Tunisie/19. VI. 2004//leg.: R. Denis &/G. Miessen/collection: G. Miessen” (PCRK); one male, “Udna/Tunis bor./Exp. Obenb.” (NMPC); one male, “Tunisia 20. 5. 95/Zaghouan mts./Krajčík M. leg.//coll. general/National Museum/Prague, Czech Republic“ (NMPC); one male, “NW Tunisia, Le Kef prov./8 km NW of Le Kef/1. VI. 2005/S. Kadlec lgt.//ex coll. S. Kadlec/National Museum/Prague, Czech Republic” (NMPC); one male, “Kairouan/Tunisia, G.C.C.//G. C. Champion/BMNH (E) 1927-409” (BMNH); one male, “Kairouan/Tunis/Exp. Obenb.” (NMPC); one male, “Tunisia/Maxula-Radis//*Malacogaster*/*bassii* Luc ?/det. W. Wittmer” (HNHM); one male, “Tunisia NE, 29. 5./Menzel Bouzelfa 20km E/Lgt. F. Houška 2008” (PCRK). **Spain (Iberian Peninsula)**. One male, “Valencia/Hispania//Torrente/15-V-1904//*Malacogaster*/*nigripes*” (NMPC); four males, “Hisp.: 1. vi—14. vi/Costa del Azahar/Oropesa de Mar/J. Macek lgt. 1991//coll. general/National Museum/Prague, Czech Republic” (NMPC); one male, “Elche/IV 05//Spanien//M.//coll. Stierlin//Pic determ.//*Malacogaster*/*nigripes* Schf.” (SDEI); one male, “Spanien//coll. Fran-/klin Müller.//*Malacogaster*/*nigripes* Schauf./det. R.Hicker” (SDEI); two males, “Col del Sr. Perez Arcas” (MNCN); one male, “Valencia (Hispania) Moróder//M.N.C.N. Madrid//*Malacogaster passerinii* Bassi, 1832 [sic!], P. Bahillo det. 2003” (MNCN); one male, “Valencia, Hispania, F. Moroder//M.N.C.N. Madrid//*Malacogaster passerinii* Bassi, 1832 [sic!], P. Bahillo det. 2003” (MNCN). **Spain (Mallorca)**. One male, “Mallorca/14. 4. 83//Friese//*Malacog*./*Passerinii*” (NMPC); one male, “Mallorca/14. 4. 83/Friese//*nigripes* Schauf/Coll. Reitter” (HNHM); one male, “Mallorca/3. 5. 83//Friese” (NMPC).

**Differential diagnosis**. This species is characterized by the relatively small eyes, with the minimal interocular distance 2.40–2.70 times maximum eye diameter, the pronotum distinctly narrower anteriorly than posteriorly, gradually widened towards posterior margin, and the elytral pubescence usually yellowish to reddish brown. Typical specimens also have bicolored tibiae, basally dark brown and then gradually lighter towards the apex. Morphologically similar *M. nigripes* (Figure 12, Figure 13 and Figure 14) differ in the darker coloration of elytral pubescence (dark reddish brown to black) and the tibia always uniformly dark brown to black (but see Remarks under *M. nigripes*). *Malacogaster theyri* (Figure 22) from Morocco, which has the similar shape of pronotum, differs from *M. passerinii* in having large eyes, with their minimal frontal separation 1.85 times maximum eye diameter. Morphologically similar *M. maculiventris* (Figure 9, Figure 10 and Figure 11) differs in having relatively larger eyes, with the minimal interocular distance 1.85–2.30 times the maximum eye diameter, the pronotum subquadrate with subparallel to slightly rounded sides, with width at posterior angles 1.00–1.10 times width at anterior angles (1.15–1.30 times in *M. passerinii*), and parameres relatively wider at apex in a dorsal view (subapically distinctly narrowed in dorsal view in *M. passerinii*).

**Diagnostic redescription**. Based on the material listed above. Male. Body (Figure 15a,b,d,e, Figure 16a–c, Figure 17a–c and Figure 18a–h) 4.30–8.50 mm long, 2.65–2.95 times as long as wide; antennae brown to dark brown, scape and pedicel dark brown, head dark brown to black, pronotal disk and hypomeron yellowish to reddish brown, scutellum dark brown to black, elytra dark brown to black, thorax underside brown to black, legs with coxae partly dark brown, apically yellowish brown, femora dark brown, tibiae usually basally dark brown and then gradually lighter towards apex or entirely reddish brown to dark brown, tarsi yellowish brown, abdominal ventrites yellowish to reddish brown, sometimes with basal ventrites medially darker. Body pubescence long, yellowish to reddish brown. Head 1.00–1.15 times as wide as anterior margin of pronotum, and 0.80–0.95 times as wide as pronotum measured at widest place. Fronto-clypeal region (Figure 3a,b, Figure 16e,f and Figure 17d) short and wide, apically widely concave; eyes relatively small, their minimum frontal separation 2.40–2.70 times maximum eye diameter; labrum large, subtrapezoidal, well visible, anteriorly slightly concave; antenna (Figure 3g) with antennomere III about 1.15–1.35 times longer than antennomere IV; median antennomeres 1.15–1.45 times as wide as long. Pronotum (Figure 4a, Figure 15c, Figure 16g and Figure 17e) subtrapezoidal, 1.20–1.35 times as wide as long when measured at widest place, narrowest at one third after anterior angles, widest posteriorly, with lateral sides slightly bisinuate; elytra (Figure 4g, Figure 15a,d and Figure 16a) elongate, combined 1.75–2.10 times as long as wide, and 2.85–3.60 times as long as pronotal length. Abdominal sternite IX 2.15–2.30 times as long as wide; tergite X 1.85–2.00 as long as wide (Figure 5c–e, Figure 16h–j and Figure 17f–h). Aedeagus (Figure 5f–h, Figure 16k–m and Figure 17i–k) about 1.90–2.15 as long as wide; median lobe 1.20–1.30 times as long as phallobase, and 2.30–2.65 times as long as lateral portion of paramere; paramere elongate, partly membranous apically, with distinct latero-apical projection on inner side, subapically more or less distinctly narrowed in dorsal view, apex truncate, usually distinctly concave in lateral view; phallobase 0.55 times as long as whole aedeagal length, 1.05–1.15 times as long as wide, and 1.80–2.20 times as long as lateral portion of paramere.

**Variability**. This species is 4.30–8.50 mm long. The eyes are relatively small, their minimum frontal separation is 2.40–2.70 times the maximum eye diameter. The shape of the pronotum is slightly variable; it is 1.20–1.35 times as wide as long when measured at widest place, and also more or less subtrapezoidal, with width at posterior angles 1.15–1.30 times width at anterior angles, and with posterior angles more or less produced. The elytral pubescence varies from yellowish through light brown to reddish brown, in several specimens from northern Africa it is slightly darker. The relative length of the parameres also varies in this species.

**Distribution**. Europe: Italy (Sardinia, Sicily, and surrounding smaller islands), Spain (Iberian Peninsula, Balearic Islands), Portugal [specimens from the Balearic Islands and Portugal were not examined by us; for more information see [35] and references therein]; Africa: Algeria, Morocco, Tunisia (Figure 25d). This species was recorded from several localities in Morocco (e.g., [92]) but the identity of the Moroccan specimens is questionable and needs further investigation [34,48]. The only specimen from Morocco we were able to study is the lectotype of *M. olcesei* from Tangier.

**Literature**. Dejean (1833: 104): *Ctenidion thoracicum* and *C. ruficollis* [published without description] [21]; Bassi (1834: pl. 99): original description, drawings of male habitus dorsal and lateral, head, abdomen, antenna, leg [18]; Dejean (1837: 117): *Ctenidion thoracicum* and *C. ruficollis* [published without description] [66]; Sturm (1843: 77): checklist; *Ctenidion thoracicum* and *C. ruficollis* [published without description] [68]; Lucas (1847: 185): catalogue [70]; Rosenhauer (1856: 140): remark [71]; Redtenbacher (1858: 525): catalogue, original description of *M. passerinii* var. *thoracica* [20]; Desmarest (1860: 8): remark [73]; Jacquelin du Val (1860: 164): catalogue, drawing of male habitus [74]; Wollaston (1864: 215): comparison with other species [22]; Wollaston (1865: 193): comparison with other species [75]; Kiesenwetter (1866: 244): distributional remark [76]; Schaufuss (1867: 86): comparison with other species [*M. passerinii* var. *thoracicus* [sic!] erroneously attributed to Rossi] [23]; Gemminger (1869: 1684): catalogue [also as *M. passerinii ruficollis* (nomen nudum), and as *M. passerinii* var. *thoracica*] [77]; Baudi di Selve (1871: 62): comparison with other species [26]; Lucas (1871: 23): comparison with other species [25]; Marseul (1873: 414): catalogue, comparison with other species [79]; Redtenbacher (1873: 19): catalogue [80]; Bertolini (1874: 132): catalogue [as *Malacoguster*; sic!; also as *M. ruficollis* and *M. thoracicus*; sic!] [64]; Oliveira (1884: 190): catalogue [82]; Failla-Tedaldi (1887: 159): remark [84]; Ragusa (1893: 358): catalogue [also as var. *thoracica*] [86]; Reitter (1894: 4): identification key [as *M. passerini*; sic!; also as var. *thoracica*] [6]; Fairmaire (1895: cx): comparison with other species [27]; Bertolini (1900: 71): catalogue [also as var. *thoracica*] [88]; Xambeu (1901: 37): larva description [as *M. passerini*; sic!] [89]; Bourgeois (1904: 481): comparison with other species [90]; Olivier (1910: 4): catalogue [also as *M. passerinii* var. *thoracicus;* sic!] [8]; Escalera (1914: 225): catalogue [92]; Cros (1925: 302): comparison with other species, larval remark [also as *M. pesserinii*; sic!] [30]; Dodero (1925: 7): comparison with other species [29]; Seurat (1925: 285): remark [96]; Winkler (1925: 522): catalogue [also as *M. passerinii* var. *thoracicus*; sic!] [14]; Cros (1926: 198): remark, drawing of larval abdomen [97]; Luigioni (1929: 616): catalogue [also as *thoracicus*] [98]; Porta (1929: 47): catalogue [also as var. *thoracica*; sic!] [99]; Cros (1930: 133): biology, redescription, larva description, comparison with other species, drawings of larva abdominal apex and larva mouth parts [4]; Gridelli (1930: 97): catalogue, remark [100]; Fuente (1931: 64): catalogue, distribution [101]; Balduf (1935: 101): larva, biology [as *M. passerini*; sic!] [103]; Clausen (1940: 544): remark [105]; Seabra (1943: 64): catalogue [107]; Wittmer (1944: 204): catalogue [as *M. passerini*; sic!, also as var. *thoracicus*; sic!] [9]; Pardo Alcaide (1945: 459): remark [as *M. passerini*; sic!] [31]; Peyerimhoff (1949: 265): comparison with other species [32]; Pic (1951: 296): remarks, original descriptions of *M. notativentris* and *M. olcesei* [33]; Kocher (1956: 24): catalogue [also as *M. olcesei*] [34]; Goidanich (1957: 565): remark [also as *M. passerinii* var. *thoracica*] [111]; Gridelli (1960: 386): remark [112]; Torres Sala (1962: 239): catalogue, distribution [113]; Kocher (1964: 44): catalogue [114]; Magis (1966: 464): remark on larva [115]; Kocher (1969: 43): catalogue [116]; Baronio (1974: 175): remark [117]; Poggi (1995: 6): checklist [121]; Sparacio (1997: 65): catalogue, distribution, drawing of male habitus [122]; Poggi (2003: online): catalogue [124]; Bahillo de la Puebla and López Colón (2005: 124): revision, identification key, distributional map, photographs of male habitus and antenna [35]; Bocak (2007: 210): catalogue, var. *thoracicus* [sic!] as a synonym of *M. passerinii* [15]; Faucheux and Agnas (2008: 109): hypermetamorphosis, larva and female description, distributional map, drawing of larval cerci, photographs of larvae and females [38]; Löbl and Smetana (2010: 25): catalogue [as *M. notativentris* and *M. olcesei*] [36]; Faucheux and Agnas (2011: 79): biology (larvae and females), photographs of female mouthparts [39]; Zapata de la Vega and Sánchez-Ruiz (2012: 125): catalogue, distributional maps, *M. thoracinus* [sic!] as synonym of *M. passerinii* [56]; Zapata de la Vega and Sánchez-Ruiz (2013: 180): catalogue [131]; Faucheux and Agnas (2014: 258): remark [132]; Faucheux and Kundrata (2014: 97): larval characteristics on the antennae of neotenic females, photograph and drawing of female antenna [133]; Kundrata et al. (2014: 167): molecular phylogeny [11]; Kundrata et al. (2014: 458): remark [57]; Zapata de la Vega and Sánchez-Ruiz (2014: 157): catalogue [134]; Faucheux (2015: 57): remark on larva [135]; Faucheux (2015: 75): remark on biology [136]; Faucheux (2015: 188): remark [137]; Kobieluszova and Kundrata (2015: 91): female antennal morphology [138]; Zapata de la Vega and Sánchez-Ruiz (2015: 186): catalogue [142]; Baalbergen et al. (2016: 168): remark on larva [5]; Bocak et al. (2016: Supplementary Materials): molecular phylogeny [143]; Faucheux (2016: 209): remark, identification problem [42]; Faucheux (2016: 221): remark on biology [43]; Faucheux (2016: 230): antennal morphology, photographs of male and female anntennal morphology and male habitus [44]; Faucheux (2016: 271): remark, identification problem [144]; Faucheux (2016: 288): remark, identification problem [45]; Faucheux (2016: 311): male antennal morphology, comparison with other species, identification problem [46]; Faucheux and Agnas (2016: 60): hypermetamorphosis, photographs of male and female immature stages [47]; Faucheux and Ballardini (2016: 187): remark, identification problem [48]; Faucheux and Beaulieu (2016: 102): hypermetamorphosis, photographs of hypermetamorphosis in male and female, habitus of male and female larvae [49]; Faucheux and Beaulieu (2016: 107): primary larva antennae and associated sensilla, photographs and/or drawings of primary larva head and antenna [50]; Faucheux and Beaulieu (2016: 149): antennal sensilla in the male imago, photographs of male head, pronotum and antenna [145]; Faucheaux et al. (2016: 121): mouthparts and associated sensilla in primary larva, photographs of primary larva habitus, head, antenna, and mouthparts [51]; Faucheaux et al. (2016: 1): larval and adult morphology, ecology, distributional map, comparison with other species, photographs of immature stages, and male and female habitus [146]; Kundrata et al. (2016: 296): molecular phylogeny [147]; Zapata de la Vega and Sánchez-Ruiz (2016: 178, 197): catalogue [148]; Faucheux (2017: 1): taxonomic remark, mouthparts, remarks [52]; Faucheux (2017: 17): remark on biology [149]; Faucheux (2017: 1): taxonomic remark, remark on biology [53]; Faucheux (2017: 1): taxonomic remark [150]; Faucheux and Kundrata (2017: 106): antennal morphology, taxonomic remark, photographs of male habitus, antennal morphology [16]; Zapata de la Vega and Sánchez-Ruiz (2017: 275): catalogue [151]; Faucheux (2018: 1): taxonomic remark [54]; Faucheux and Agnas (2018: 1): taxonomic remark [55]; Kundrata et al. (2018: suppl.): molecular phylogeny [as *M. passerini*; sic!] [152]; Lequet and Faucheux (2018: 14): remark on breeding [153]; Sormova et al. (2018: 2): molecular phylogeny [155]; Zapata de la Vega and Sánchez-Ruiz (2018: 31): catalogue [156]; Bi et al. (2019: 82): molecular phylogeny [157]; Kundrata and Bocak (2019: 418/441): molecular phylogeny, review [also as *M. olcesei*], photographs of male pronotum, mesoventrite and abdomen [1]; Kundrata et al. (2019: 100): generic catalogue [158]; Zapata de la Vega and Sánchez-Ruiz (2019: 74): catalogue [160]; Lo Valvo (2020: 170): checklist [162]; Valcárcel and Prieto Piloña (2020: 317): remark [58]; Dal Cortivo et al. (2021: 20/69): key, checklist, biology, drawings of antennae, photograph of male habitus [163]; Poggi (2021: online): catalogue [164].

**Remarks**. Cros [4] already proposed that *M. passerinii* is probably conspecific with *M. nigripes* which, according to him, differs mainly in the coloration of the pronotum, legs, and elytral pubescence (see also [35]). We found minimal differences in the body coloration and morphology of these species, and it is possible that they actually represent a single species. We prefer to keep them as separate species until more detailed research, including the DNA approach, is available.

We here synonymize *Malacogaster notativentris* Pic, 1951 and *M. olcesei* Pic, 1951 with *M. passerinii*. The holotype of *M. notativentris* shares coloration and morphology with typical *M. passerinii*. They share partly reddish brown tibiae and elytral pubescence, relatively small eyes, a prototum that is clearly widest at posterior angles, and similar male genitalia (Figure 16 and Figure 18). Moreover, *M. notativentris* was collected in Philippeville [now Skikda], Algeria, which is not far from Sardinia (type locality of *M. passerinii*). Lectotype of *M. olcesei* is placed within the wider concept of *M. passerinii* based on the small eyes, coloration of elytral pubescence, and shapes of the pronotum and male genitalia (Figure 17 and Figure 18).

Specimens from Oualidia, Morocco which were in earlier studies by Faucheux and colleagues identified as *M. passerinii* (e.g., [38,39,51]), were later re-determined as *M. nigripes* based on the coloration of legs in an adult male [48]. It is, however, possible that the specimens from Oualidia represent *M. maculiventris* (see Remarks under the latter species for more information). 

#### 3.1.6. *Malacogaster rubripes* Peyerimhoff, 1949

**Figure 19:**
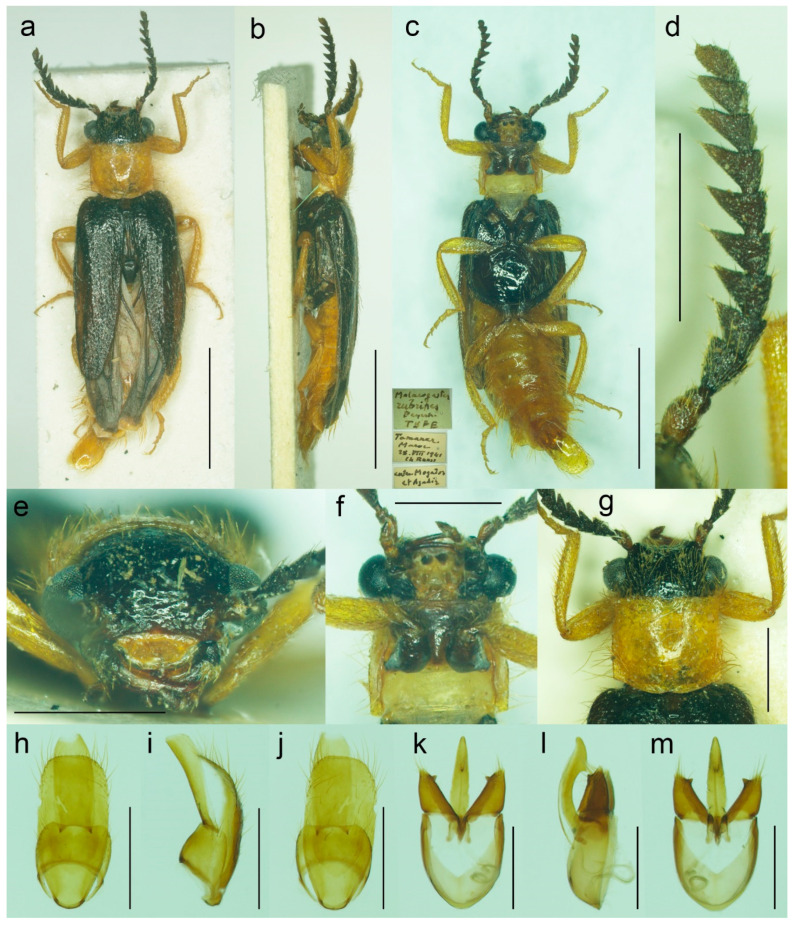
*Malacogaster rubripes* Peyerimhoff, 1949, male lectotype. (**a**) Habitus, dorsal view; (**b**) habitus, lateral view; (**c**) habitus, ventral view; (**d**) right antenna; (**e**) head, frontal view; (**f**) head and prothorax, ventral view; (**g**) pronotum, dorsal view; (**h**) genital capsule formed by tergites IX and X, and sternite IX, dorsal view; (**i**) genital capsule formed by tergites IX and X, and sternite IX, lateral view; (**j**) genital capsule formed by tergites IX and X, and sternite IX, ventral view; (**k**) aedeagus, dorsal view; (**l**) aedeagus, lateral view; (**m**) aedeagus, ventral view. Scale bars = (**a**–**c**) 2.5 mm; (**d**–**j**) 1.0 mm; (**k**–**m**) 0.5 mm.

(Figure 19 and Figure 25a)

*Malacogaster rubripes* Peyerimhoff, 1949: 265 [32].

**Type material**. Described based on two male specimens. Lectotype by present designation, male, “Tamanar./Maroc/28. VIII 1941/Ch Rungs//entre Mogador [Essaouira]/et Agadir//*Malacogaster*/*rubripes*/Peyerh./TYPE” (MNHN).

**Type locality**. Morocco: Essaouira Prov., Tamanar.

**Other material examined**. **Morocco**. One male, “Morocco, Teima, 1 km W/Tamelalt, ca 10 km NW/Ouled, 78 m, 23.5.2013/Z. Lucbauer leg.” (PCRK); one male, “12.V.2015/Ait Maala, 700 m/NE Oulad Berhil/Morocco/lgt. Z. Lucbauer//BMNH{E}/2018-80.” (BMNH). Kocher [34] mentioned an additional specimen from Inezgane near Agadir, and there is also one specimen from the High Atlas Mts. (6 km W Taliouine, 980 m, 30°33′00′′ N, 07°58′50′′ W, 29.04.2012, collected at flight) in the collection of F. Houška, Czech Republic (PCFH).

**Differential diagnosis**. This species can be easily recognized by its large eyes (their minimum frontal separation 1.90 times the maximum eye diameter), the head including eyes distinctly wider than the pronotum when measured at the widest place, and legs (except coxae) yellowish brown (Figure 19a,c). *Malacogaster rutllanti* is the only other species with light colored legs but it differs by much smaller eyes, a head including eyes not wider than the pronotum when measured at the widest place, and pronotum distinctly gradually widened posteriorly (Figure 21c–h).

**Diagnostic redescription**. Based on the lectotype. Male. Body (Figure 19a–c) 5.60 mm long, 2.80 times as long as wide; dark brown to black, prosternum posteriorly lighter, labrum, ventral portion of head, pronotum including hypomeron, and legs except coxae yellowish brown, abdomen yellowish to reddish brown. Body pubescence yellowish brown, setae on elytra yellowish to reddish brown. Head 1.15 times as wide as anterior margin of pronotum, and 1.10 times as wide as pronotum measured at widest place. Fronto-clypeal region (Figure 19e) short and wide, apically widely concave; eyes relatively large, their minimum frontal separation 1.90 times maximum eye diameter; labrum large, subtrapezoidal, well visible, anteriorly slightly concave; antenna (Figure 19d) with antennomere III about 1.30 times longer than antennomere IV; median antennomeres about 1.35 times as wide as long. Pronotum (Figure 19g) subquadrate, 1.15 times as wide as long when measured at widest place, narrowest just behind anterior angles, widest medially, with lateral sides slightly bisinuate; prosternum (Figure 19f) about 1.85 times as wide as long medially; elytra (Figure 19a) relatively short, combined 1.90 times as long as wide, and 3.30 times as long as pronotal length. Abdominal sternite IX about 2.15 times as long wide; tergite X very long, 2.25 times as long as wide (Figure 19h–j). Aedeagus (Figure 19k–m) 2.05 as long as wide; median lobe relatively robust, 1.10 times as long as phallobase, and 2.50 times as long as lateral portion of paramere; paramere robust, subtruncate apically, with latero-apical projection on inner side, apically slightly emarginate at lateral view; phallobase robust, 0.55 times as long as whole aedeagal length, 1.15 times as long as wide, and 2.25 times as long as lateral portion of paramere.

**Variability**. The non-type specimen from Teima is 6.20 mm long and has the prosternum slightly darker and procoxae slightly lighter than the lectotype. Additionally, it has the pronotum slightly more transverse, 1.20 times as wide as long when measured at widest place, widest at posterior angles (but almost as wide as medially). Its parameres are apically less slightly emarginate at lateral view.

**Distribution**. Morocco (Figure 25a). Bocak [15] listed only Algeria (instead of Morocco) for this species but we are not aware of any specimen of *M. rubripes* from that country. All known specimens are known from southern part of Morocco.

**Literature**. Peyerimhoff (1949: 249/265): original description [32]; Pic (1951: 295): remarks [33]; Kocher (1956: 24): catalogue [34]; Bocak (2007: 210): catalogue [15]; Kundrata and Bocak (2019: 441): review [1].

#### 3.1.7. *Malacogaster ruficollis* Dodero, 1925, stat. nov.

**Figure 20:**
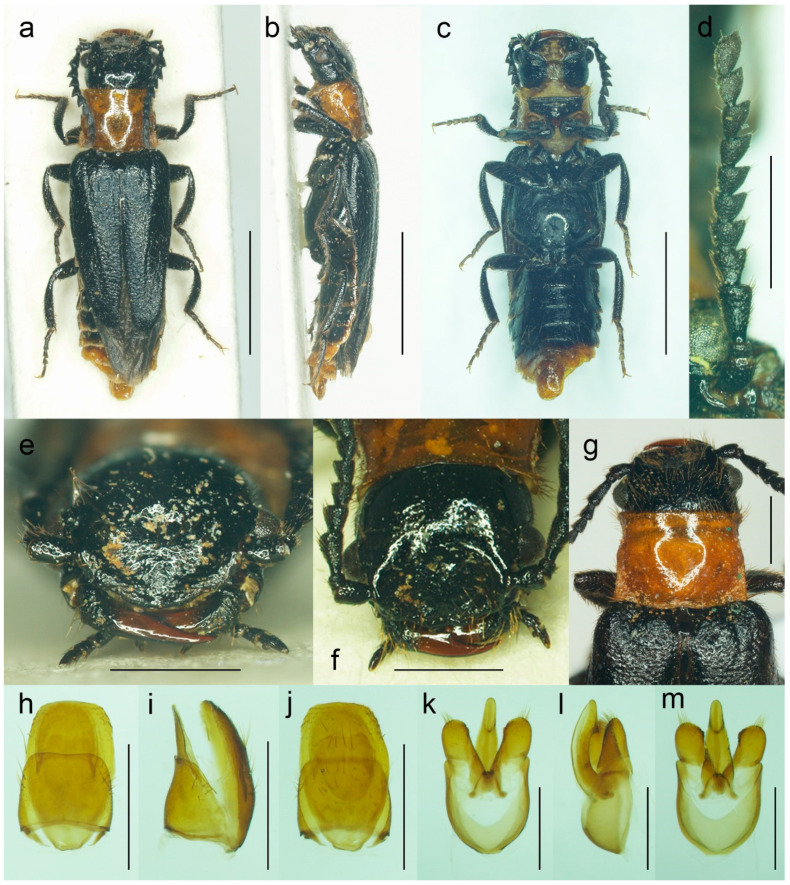
*Malacogaster ruficollis* Dodero, 1925, stat. nov., male. (**a**) Habitus, dorsal view; (**b**) habitus, lateral view; (**c**) habitus, ventral view; (**d**) left antenna; (**e**) head, frontal view; (**f**) head, fronto-dorsal view; (**g**) pronotum, dorsal view; (**h**) genital capsule formed by tergites IX and X, and sternite IX, dorsal view; (**i**) genital capsule formed by tergites IX and X, and sternite IX, lateral view; (**j**) genital capsule formed by tergites IX and X, and sternite IX, ventral view; (**k**) aedeagus, dorsal view; (**l**) aedeagus, lateral view; (**m**) aedeagus, ventral view. Scale bars = (**a**–**c**) 3.0 mm; (**d**–**j**) 1.0 mm; (**k**–**m**) 0.5 mm.

**Figure 21:**
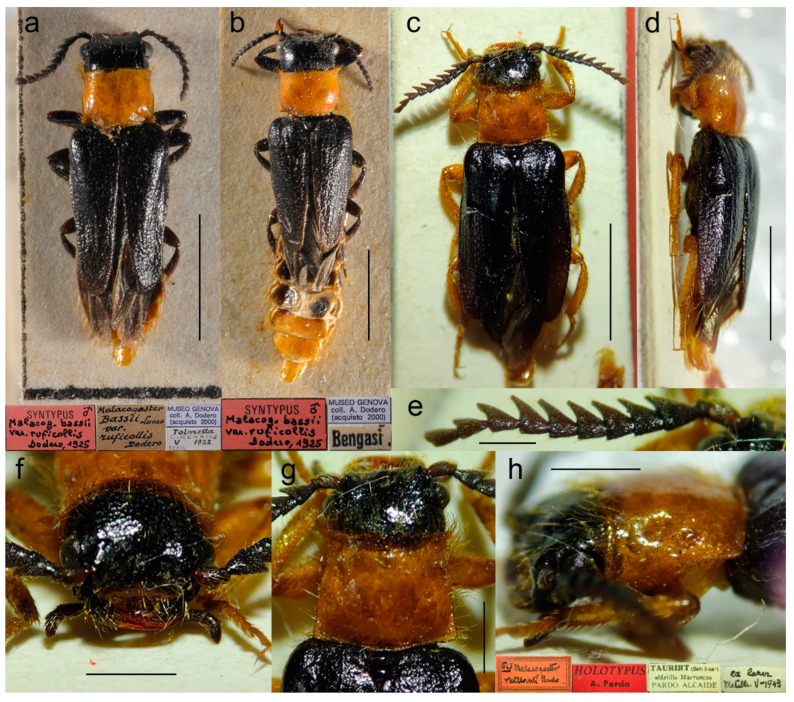
*Malacogaster ruficollis* Dodero, 1925, stat. nov., male syntypes. (**a**) Syntype from Tolmetta, habitus, dorsal view; (**b**) Syntype from Benghazi, habitus, dorsal view. *Malacogaster rutllanti* Pardo Alcaide, 1945, male holotype. (**c**) habitus, dorsal view; (**d**) habitus, lateral view; (**e**) left antenna; (**f**) head, frontal view; (**g**) pronotum, dorsal view; (**h**) pronotum, lateral view. Scale bars = (**a**,**b**) 2.5 mm; (**c**,**d**) 3.0 mm; (**e**) 0.5 mm; (**f**–**h**) 1.0 mm. Photographs of *M. ruficollis* courtesy of R. Poggi (MSNG). Photographs of *M. rutllanti* courtesy of G. O. Muñoz (MUNA).

(Figure 20, Figure 21a,b and Figure 25a)

*Malacogaster bassii* var. *ruficollis* Dodero, 1925: 7 [29].

**Type material**. Described based on an unknown number of specimens. Two syntypes found in the MSNG (identity confirmed based on the detailed photographs kindly provided by R. Poggi; Figure 21a,b): one syntype, male, “Bengasi//Syntypus ♂/*Malacog. bassii*/var. *ruficollis*/Dodero, 1925//Museo Genova/coll. A. Dodero/(acquisto 2000)” (MSNG); one syntype, male, “Tolmetta/Cirenaica/V 1922/Festa//*Malacogaster*/*Bassii* Lucas/var./*ruficollis*/Dodero//Syntypus ♂/*Malacog. bassii*/var. *ruficollis*/Dodero, 1925//Museo Genova/coll. A. Dodero/(acquisto 2000)” (MSNG). We did not designate the lectotype as the species identity is clear.

**Type localities**. Libya: Benghazi and Tolmetta.

**Material examined**. **Libya**. One male, “Libya. Benghazi/zahrada [garden]. 11. 4. 79/K. Hůrka leg.//ex coll. K. Hůrka/National Museum/Prague, Czech Republic//*Malacogaster*/*nigripes* Schauf./VI. Švihla det. 1980” (NMPC); one male, “Libya. Dj. Akhdar/N of Al Bejda/Vadi Jarjaroma/29. 4. 80, K. Hůrka//ex coll. K. Hůrka/National Museum/Prague, Czech Republic” (NMPC).

**Differential diagnosis**. This is the only species of *Malacogaster* with clypeus distinctly produced forwards and covering labrum (Figure 20e,f). All other species have clypeus short and wide, usually widely concave, and the labrum fully exposed. Additionally, this is the only species of *Malacogaster* known from Libya.

**Diagnostic redescription**. Based on the specimens listed above; body coloration and habitus, pronotum and elytra measurements were also obtained from the syntypes. Male. Body (Figure 20a–c and Figure 21a,b) 5.00–7.20 mm long (syntype from Tolmetta 5.15 mm, syntype from Benghazi 5.95 mm), 2.40–3.00 times as long as wide; dark brown to black, tarsi lighter, pronotum, hypomeron, and apical abdominal segments yellowish to reddish brown. Setae on head and pronotum yellowish brown, on legs and elytra yellowish brown to brown. Head 1.00–1.10 times as wide as anterior margin of pronotum, and 0.95–1.05 times as wide as pronotum measured at widest place. Fronto-clypeal region (Figure 20e,f) pronounced forwards and apically rounded; eyes small, their minimum frontal separation 2.85–3.00 times maximum eye diameter; labrum transverse, hidden by anteriorly expanded clypeus; antenna (Figure 20d) with antennomere III about 1.50–1.60 times longer than antennomere IV; median antennomeres about as wide as long. Pronotum (Figure 20g) subquadrate, 1.25–1.35 times as wide as long when measured at widest place, narrowest at anterior third just before anterior angles, widest posteriorly or subequally posteriorly and medially (one non-type specimen with less produced posterior angles widest medially), with lateral sides bisinuate; elytra (Figure 20a and Figure 21a,b) relatively short, combined 1.55–1.85 times as long as wide, and 2.65–3.15 times as long as pronotal length. Abdominal sternite IX about 1.65 times as long as wide; tergite X not elongate, 0.95 times as long as wide (Figure 20h–j). Aedeagus (Figure 20k–m) 1.80–1.85 times as long as wide; median lobe robust, 1.05–1.10 times as long as phallobase, and 2.40 times as long as lateral portion of paramere; paramere robust, apically sclerotized and rounded, with only slight traces of latero-apical projection on inner side; phallobase robust, 0.60 times as long as whole aedeagal length, 1.05–1.10 times as long as wide, and 2.15–2.35 times as long as lateral portion of paramere.

**Variability**. This species shows the intraspecific variability in the body size (length 5.00–7.20 mm) and proportions (body 2.40–3.00 times as long as wide, combined elytra 1.55–1.85 times as long as wide). Head is either as wide as or slightly wider than the anterior margin of pronotum. Most specimens have brown elytral pubescence; however, one specimen is generally paler and has yellowish brown setae on the elytra. Although most specimens have a pronotum widest posteriorly or subequally posteriorly and medially, one non-type specimen has less produced posterior angles so that its pronotum is widest medially.

**Distribution**. Libya (Cyrenaica) (Figure 25a).

**Literature**. Dodero (1925: 7): original description [as *M. bassii* var. *ruficollis*] [29]; Gridelli (1930: 97): catalogue, remark [100]; Wittmer (1944: 204): catalogue [as *M. bassii* var. *ruficollis*] [9]; Gridelli (1960: 386): remark [as *M. bassii ruficollis*] [112]; Bocak (2007: 210): catalogue [as a synonym of *M. bassii*] [15].

**Remark**. This species was originally described as a variety of *M. bassii* Lucas, 1870 [24,25], and Bocak [15] de facto synonymized it under *M. bassii* without any supporting evidence. It should be noted that the type series or any other reliably identified material of *M. bassii* is unavailable for study. That species was described based on a large male specimen from Algeria (8.50 mm) with an obviously darker median portion of pronotal disk, thus we consider it a different species, and treat *M. ruficollis* as a separate species here. 

Zanon [7] reported *M. nigripes* from the Cyrenaica region of Libya; however, this record probably belongs to *M. ruficollis* which has also dark legs and is known only from that area.

#### 3.1.8. *Malacogaster rutllanti* Pardo Alcaide, 1945

(Figure 21c–h and Figure 25a)

*Malacogaster rutllanti* Pardo Alcaide, 1945: 457 [31].

**Type material**. Holotype, male, “Taurirt [Taourirt] (Beni Sicar)/Melilla-Marruecos/Pardo Alcaide//Holotypus/A. Pardo//567 *Malacogaster*/*rutllanti* Pardo” [additional information from the original description: V-1943 (ex larva) [31]] (MUNA). Holotype was not examined by us; however, we had at our disposal detailed photographs kindly provided by Gloria Ortega Muñoz (MUNA) (see Acknowledgements).

**Type locality**. Morocco: Taourirt (Beni Sicar).

**Differential diagnosis**. This species is the most similar to *M. rubripes* with which it shares the uniformly light colored legs (yellowish to reddish brown in *M. rutllanti*, yellowish brown in *M. rubripes*; Figure 19a–c). *Malacogaster rutllanti* differs from *M. rubripes* by much smaller eyes, head including eyes not wider than pronotum when measured at widest place, and pronotum distinctly gradually widened posteriorly (Figure 19g).

**Diagnostic redescription**. Male (holotype). Body (Figure 21c,d) approximately 7 mm long, 2.65 times as long as wide; dark brown to black, antennae towards apex slightly lighter, labrum, ventral portion of head, pronotum including hypomeron, legs except coxae, and abdomen yellowish to reddish brown. Head 1.05 times as wide as anterior margin of pronotum, and 0.90 times as wide as pronotum measured at widest place. Fronto-clypeal region (Figure 21f) short and wide, apically widely concave; eyes relatively small; labrum large, subtrapezoidal, well visible, anteriorly slightly concave; antenna (Figure 21e) with antennomere III approximately 1.3 times longer than antennomere IV; median antennomeres approximately 1.5 times as wide as long. Pronotum (Figure 21g) subtrapezoidal, 1.25 times as wide as long when measured at widest place, narrowest at one third after anterior angles, widest posteriorly, with lateral sides slightly bisinuate, at posterior half almost straight; elytra (Figure 21c) relatively short, combined 1.80 times as long as wide, and 3.00 times as long as pronotal length. Aedeagus (Figure 2a in [31]) with paramere robust, subtruncate apically, with latero-apical projection on inner side.

**Distribution**. Morocco (Figure 25a).

**Literature**. Pardo Alcaide (1945: 457): original description, drawings of male habitus and genitalia [31]; Wittmer (1948: 115): catalogue [108]; Pic (1951: 295): remarks [33]; Kocher (1956: 25): catalogue, synonymization with *M. parallelocollis* [34]; Bahillo de la Puebla & López Colón (2005: 124): remark [35]; Bocak (2007: 210): catalogue [15]; Kundrata & Bocak (2019: 441): review [1]; Ortega (2019: 340): type material information [159]; Chavanon (2020: 69): catalogue [161].

**Remark**. Kocher [34] synonymized *M. rutllanti* with *M. parallelocollis* without any explanation. Subsequent authors did not follow Kocher’s synonymization. *Malacogaster parallelocollis* is here synonymized under *M. maculiventris*. This species clearly differs from *M. rutllanti* by its dark brown to black legs (yellowish to reddish brown in *M. rutllanti*), pronotum width at posterior angles 1.00–1.10 times as pronotum width at anterior angles (1.15 times in *M. rutllanti*), and by apparently larger eyes. Therefore, we treat *M. rutllanti* as a different species from *M. parallelocollis*/*M. maculiventris*.

#### 3.1.9. *Malacogaster theryi* Pic, 1951

**Figure 22:**
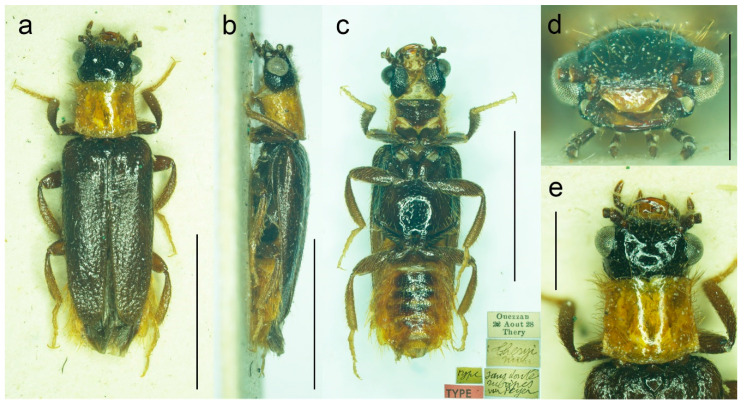
*Malacogaster theryi* Pic, 1949, male holotype. (**a**) Habitus, dorsal view; (**b**) habitus, lateral view; (**c**) habitus, ventral view; (**d**) head, frontal view; (**e**) pronotum, dorsal view. Scale bars = (**a**–**c**) 3.0 mm; (**d**,**e**) 1.0 mm.

(Figure 22 and Figure 25a)

*Malacogaster theryi* Pic, 1951: 297 [33].

**Type material**. Holotype, male, “Ouezzan [Ouazzane]/22 Aout 28/Thery//type//*theryi*/mihi//saus doute/*rubripes*/var Peyer//TYPE [red printed label]” (MNHN).

**Type locality**. Morocco: Ouazzane.

**Differential diagnosis**. This species shares relatively large eyes (i.e., their minimum frontal separation less than 2.30 times maximum eye diameter) with *M. holomelas*, *M. rubripes*, and widely defined *M. maculiventris*. It differs from *M. holomelas* (Figure 8) in having yellowish to reddish brown pronotum which is clearly widest at posterior angles (dark brown to black pronotum which is widest both medially and posteriorly in *M. holomelas*), and from *M. rubripes* (Figure 19) in having dark brown femora and parts of tibiae (yellowish brown in *M. rubripes*) and less transverse pronotum which is anteriorly about as wide as long (about 1.15 times as wide as long in *M. rubripes*). This species resembles *M. maculiventris* (Figure 9, Figure 10 and Figure 11) in a small body, large eyes and overall appearance and coloration but it differs in having a pronotum gradually widened posteriorly, with concave sides (Figure 9f).

**Diagnostic redescription**. Based on the holotype. Male. Body (Figure 22a–c) 5.50 mm long, 3.05 times as long as wide; antennomeres III–XI missing, scape and pedicel dark brown, head dark brown to black, pronotal disk and hypomeron yellowish to reddish brown, scutellum dark brown to black, elytra brown to dark brown, thorax underside dark brown, legs with coxae mostly brown, only apically yellowish brown, femora brown, tibiae light brown, tarsi yellowish brown, abdominal ventrites 1–5 dark brown medially and lighter laterally, remaining ventrites yellowish brown. Body pubescence long, yellowish. Head 1.25 times as wide as anterior margin of pronotum, and 1.10 times as wide as pronotum measured at widest place. Fronto-clypeal region (Figure 22d) short and wide, apically widely concave; eyes large, their minimum frontal separation 1.85 times maximum eye diameter; labrum large, subtrapezoidal, well visible, anteriorly slightly concave. Pronotum (Figure 22e) subtrapezoidal, 1.15 times as wide as long when measured at widest place, narrowest at one third after anterior angles, widest posteriorly, with lateral sides concave; elytra (Figure 22a) elongate, combined 2.10 times as long as wide, and 3.40 times as long as pronotal length. Pregenital segments and aedeagus missing.

**Distribution**. Morocco (Figure 25a).

**Literature**. Pic (1951: 297): original description [33]; Kocher (1956: 24): catalogue, synonymy under *M*. *olcesei* [34]; Löbl and Smetana (2010: 25): catalogue [36]; Kundrata and Bocak (2019: 441): review [1].

**Remarks**. Kocher [34] synonymized this species with *M. olcesei* without any explanation. However, *M. theyri* differs from *M. olcesei* (currently synonymized under *M. passerinii*) in having much larger eyes (their minimum frontal separation 1.85 times the maximum eye diameter versus 2.65 times in *M. olcesei*) and head including eyes distinctly wider than pronotum when measured at widest place (about as wide as pronotum in *M. olcesei*; Figure 17e). Since this species strongly resembles *M. maculiventris* in many aspects (but has a pronotum widened posteriorly and with concave sides) and also *M. passerinii* and *M. nigripes* (but has much larger eyes), further material is needed to understand its status. What is more, the only known specimen lacks the pregential segments and genitalia.

#### 3.1.10. *Malacogaster tilloides* Wollaston, 1864

**Figure 23:**
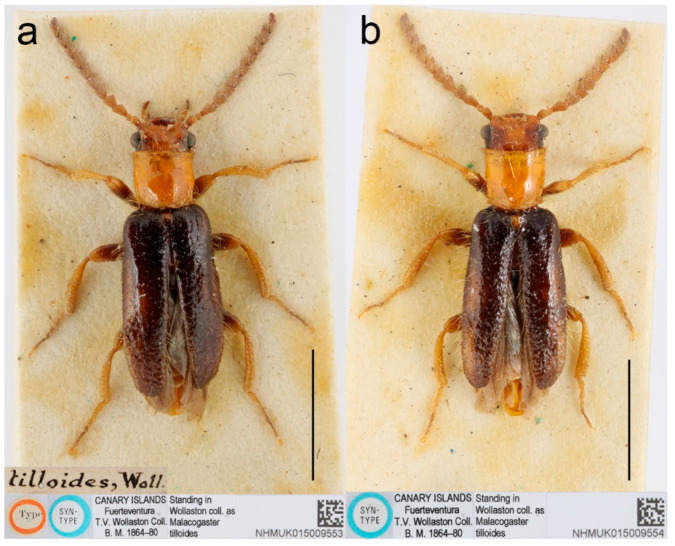
*Malacogaster tilloides* Wollaston, 1864, male syntypes (BMNH). (**a**,**b**) Habitus, dorsal view. Scale bars = (**a**) 3.0 mm; (**b**) 2.5 mm. Photographs courtesy of K. Matsumoto (BMNH).

**Figure 24:**
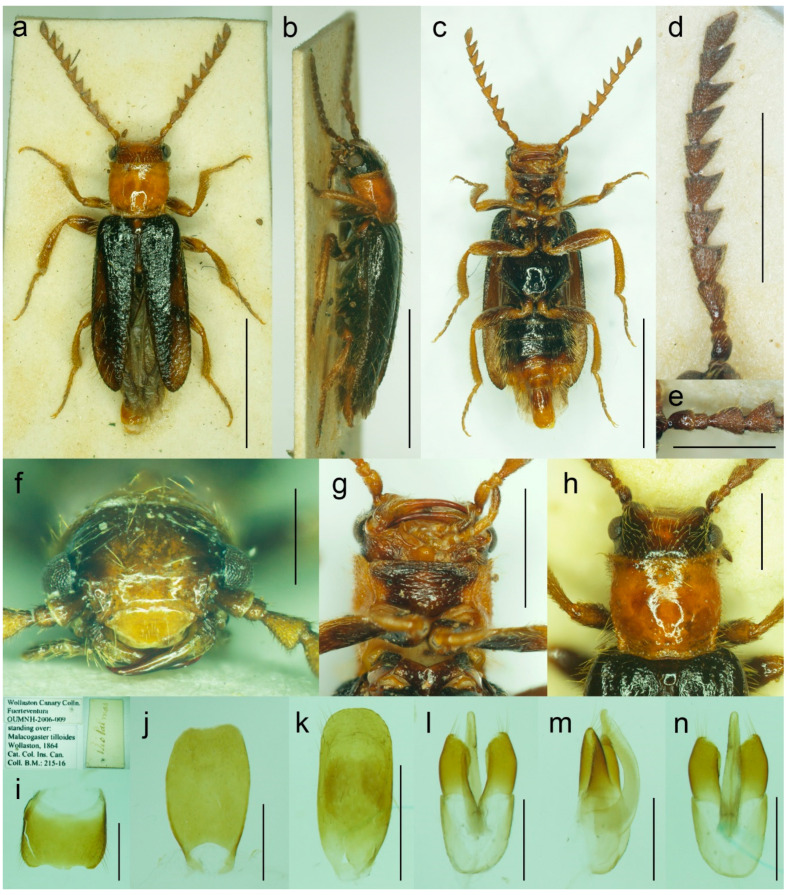
*Malacogaster tilloides* Wollaston, 1864, male syntype (OUMNH). (**a**) Habitus, dorsal view; (**b**) habitus, lateral view; (**c**) habitus, ventral view; (**d**) left antenna; (**e**) basal antennomeres of right antenna; (**f**) head, frontal view; (**g**) head and prothorax, ventral view; (**h**) pronotum, dorsal view; (**i**) abdominal tergite IX; (**j**) abdominal tergite X; (**k**) abdominal sternite IX; (**l**) aedeagus, dorsal view; (**m**) aedeagus, lateral view; (**n**) aedeagus, ventral view. Scale bars = (**a**–**c**) 3.0 mm; (**d**) 1.5 mm; (**e**,**g**,**h**,**k**) 1.0 mm; (**f**,**i,j**,**l**–**n**) 0.5 mm.

**Figure 25:**
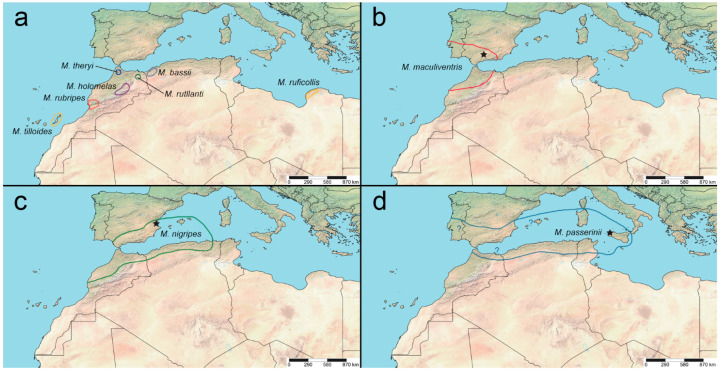
Distribution of *Malacogaster* Bassi, 1834. (**a**) *M. bassii* Lucas, 1870, *M. holomelas* Peyerimhoff, 1949, *M. rubripes* Peyerimhoff, 1949, *M. ruficollis* Dodero, 1925, *M. rutllanti* Pardo Alcaide, 1945, *M. theryi* Pic, 1951, and *M. tilloides* Wollaston, 1864; (**b**) *M. maculiventris* Reitter, 1894; (**c**) *M. nigripes* Schaufuss, 1867; (**d**) *M. passerinii* Bassi, 1834. Black stars (**b**–**d**) represent type localities; question marks (**d**) represent literature data not confirmed in this study.

(Figure 23, Figure 24 and Figure 25a)

*Malacogaster tilloides* Wollaston, 1864: 215 [22].

**Type material**. Described based on seven specimen, five of which we located. Three syntypes, males, “standing over:/*Malacogaster tilloides*/Wollaston, 1864/Cat. Col. Ins. Can./Coll. B. M.: 215-16//Rio Palmas//Wollaston Canary Colln./Fuerteventura/OUMNH-2006-009” (OUMNH). Two syntypes, males, “Canary Islands/Fuerteventura, T.V. Wollaston Coll./B. M. 1864–80//Standing in/Wollaston coll. as/*Malacogaster*/*tilloides*//*tilloides*, Woll. [only in one specimen]//Type [circle white label with red margin; only in one specimen]//Syntype [circle white label with blue margin]//NHMUK015009553 [printed label with QR code; NHMUK015009554 in the second specimen]” (BMNH). In this study we examined the syntypes from OUMNH. The syntypes from BMNH were examined from the detailed photographs kindly provided by Keita Matsumoto (BMNH) (Figure 23). We did not designate the lectotype as the species identity is clear.

**Type locality**. Spain: Canary Islands, Fuerteventura, Rio Palmas.

**Other material examined**. **Spain (Canary Islands)**. Two males, “Lanzarote/Umg. Haria/6. 3. 89 A. Evers” (SDEI); one male, “Isl. Canarias Esp./Fuertaventura [sic!]/Correlejo/3-8. III. 1985/H. Teunissen leg.//*Malacogaster*/*tilloïdes* Woll./det. A. Teunissen” (PCAT); one male, “Fuerteventrura, Carretera de Betancuria, 10. IV. 1934//*Malacogaster tiloides* [sic!] Woll. ♂, Cabrera det.//MNCN_Ent 169555” (MNCN); one male, “Fuerteventrura, Las Peñitas, 11. III. 1935//*Malacogaster tiloides* [sic!] Woll. ♂, Cabrera det.//MNCN_Ent 169549” (MNCN); one male, “Fuerteventrura, Rosa Ucala, 5. III. 1935//*Malacogaster tiloides* [sic!] Woll. ♀ [sic!], Cabrera det.//MNCN_Ent 169550” (MNCN); one male, “Fuerteventrura, Betancuria, 10. III. 1935//*Malacogaster tiloides* [sic!] Woll. ♂, Cabrera det.//MNCN_Ent 169551” (MNCN); one male, “Fuerteventrura, Carretera de Betancuria, 10. III. 1935//*Malacogaster tiloides* [sic!] Woll. ♀ [sic!], Cabrera det.//MNCN_Ent 169552” (MNCN); one male, “Fuerteventrura, Carretera de Betancuria, 10. III. 1935//*Malacogaster tiloides* [sic!] Woll. ♂, Cabrera det.//MNCN_Ent 169553” (MNCN); one male, “Fuerteventrura, Carreterade, Betancuria, 10. III. 1935//*Malacogaster tiloides* [sic!] Woll. ♂, Cabrera det.//MNCN_Ent 169554” (MNCN).

**Material reported by colleagues from the Canary Islands (not examined in this study)**. One male, “Islas Canarias, Fuerteventura: Barranco del Ciervo, 27/02/1990, P. Oromí leg.” (PCPO); one male, “Islas Canarias, Fuerteventura: Betancuria, 12/05/1974, P. Oromí leg. (PCPO); one male, “Islas Canarias, Lanzarote: Famara 01/05/2002, H. Contreras leg. (PCPO); one male, “Islas Canarias, Fuerteventura: Jandía, Ladera Culantrillo, 02/02/1994, R. García leg.” (PCRG); one male, “Islas Canarias, Fuerteventura: La Oliva, 26/02/2006, R. García leg. (PCRG); one male, “Islas Canarias, Fuerteventura: Caldera de Tiscamanita, 07/03/2011, R. García leg.” (PCRG); one male, “Fuerteventura, La Oliva 8-3-2014, R, García leg.” (PCRG), one male, “Lanzarote, Mirador del Río, 2-28-2019” (PCRG); one male, “Islas Canarias, Lanzarote: Barranco Elvira Sánchez, Haría, 30/04/2003, H. López leg.” (PCHL).

**Differential diagnosis**. This is the only species of *Malacogaster* known from the Canary Islands. It has a reddish brown head and yellowish to reddish brown antennomeres, while all other *Malacogaster* species have head and antennomeres distinctly darker, i.e., dark brown to black. *Malacogaster tilloides* and *M. ruficollis* are the only *Malacogaster* species with parameres not truncate or concave apically (clearly visible from lateral view) (Figure 20k–m). Apart from the characters mentioned above, *M. tilloides* differs from *M. ruficollis* from Libya e.g., in having the pronotum 1.05–1.10 times as wide as long when measured at widest place (1.23–1.35 times in *M. ruficollis*; Figure 20g).

**Diagnostic redescription**. Based on the syntypes from OUMNH. Male. Body (Figure 24a–c) 5.50–6.20 mm long, 3.10–3.25 times as long as wide; antennae yellowish brown to brown, basal half of scape always darker, apical half lighter, pedicel darker or slightly lighter, head brown, with medial portion of frons and clypeus lighter, pronotal disk and hypomeron yellowish to reddish brown, scutellum yellowish brown to brown, elytra dark brown, thorax underside brown to dark brown, legs with coxae mostly dark brown, only apically yellowish brown, femora brown, tibiae and tarsi yellowish brown, abdominal ventrites 1–5 dark brown, remaining ventrites yellowish brown. Body pubescence very long, yellowish. Head 1.05–1.10 times as wide as anterior margin of pronotum, and 1.05–1.10 times as wide as pronotum measured at widest place. Fronto-clypeal region (Figure 24f,g) short and wide, apically widely concave; eyes relatively small, their minimum frontal separation 2.90 times maximum eye diameter; labrum large, subtrapezoidal, well visible, anteriorly slightly concave; antenna (Figure 24d,e) with antennomere III about 1.10–1.15 times longer than antennomere IV; median antennomeres 1.15–1.20 times as wide as long. Pronotum (Figure 24h) subquadrate, 1.05–1.10 times as wide as long when measured at widest place, narrowest near posterior angles, widest anteriorly or medially, with lateral sides bisinuate; elytra (Figure 24a) elongate, combined 2.20–2.25 times as long as wide, and 3.25 times as long as pronotal length. Abdominal sternite IX about 2.20 times as long as wide; tergite X about 1.50 as long as wide (Figure 24i–k). Aedeagus (Figure 24l–n) about 2.20 as long as wide; median lobe robust, 1.55 times as long as phallobase, and 2.05 times as long as lateral portion of paramere; paramere elongate, partly membranous apically, without latero-apical projection on inner side, apex narrowly rounded apically; phallobase robust, 0.50 times as long as whole aedeagal length, 1.10 times as long as wide, and 1.30 times as long as lateral portion of paramere.

**Variability**. Most specimens are about 5–6 mm long; however, one specimen in MNCN is distinctly smaller (4.20 mm) and, on the other hand, one specimen is much larger and wider, being 8.20 mm long and about 2.70 times as long as wide. It has also unusually relatively wider pronotum which is 1.25 times as wide as long, and widest medially. Additionally, one of the non-type specimens has pronotum as wide as long when measured at the widest place, and larger eyes, with their minimum frontal separation 2.35 times maximum eye diameter. The available specimens differ slightly in the coloration of antennae (yellowish brown vs. brown, basal antennomeres darker vs. light) (Figure 23 and Figure 24).

**Distribution**. Canary Islands (Fuerteventura, Lanzarote) (Figure 25a).

**Literature**. Wollaston (1864: 215): original description [22]; Wollaston (1865: 193): catalogue [75]; Gemminger (1869: 1684): catalogue [77]; Marseul (1873: 413): catalogue, redescription, comparison with other species [79]; Marseul (1877: 42): catalogue [81]; Olivier (1910: 4): catalogue [8]; Winkler (1925: 522): catalogue [14]; Wittmer (1944: 204): catalogue [9]; Israelson et al. (1982: 118): catalogue [118]; Machado and Oromí (2000: 53): catalogue [123]; Bocak (2007: 210): catalogue [15]; Oromí et al. (2010: 279): catalogue [128]; Kundrata and Bocak (2019: 441): review [1].

### 3.2. Species Excluded from Genus Malacogaster Bassi, 1834

#### 3.2.1. *Malacogaster adusta* Chevrolat, 1854

*Malacogaster adustus* [sic!] Chevrolat, 1854: 433 [19].

**Remarks**. Described from Lebanon [19]. Transferred to *Drilus* by Kundrata et al. [57].

#### 3.2.2. *Malacogaster akbesiana* Fairmaire, 1895

*Malacogaster akbesiana* Fairmaire, 1895: cx [27].

**Remarks** Described from “Akbes”, Turkey [27]. Transferred to *Drilus* by Kundrata et al. [57].

#### 3.2.3. *Malacogaster rufipes* Baudi di Selve, 1871

*Malacogaster rufipes* Baudi di Selve, 1871: 62 [26].

**Remarks**. Described from Cyprus [26]. Known from Cyprus, Greece (Rhodes), Israel. Transferred to *Drilus* by Zurcher [28] (see also [57,140,155]).

#### 3.2.4. *Malacogaster truquii* Baudi di Selve, 1871

*Malacogaster truquii* Baudi di Selve, 1871: 61 [26].

**Remarks**. Described from Cyprus [26]. Transferred to *Drilus* by Zurcher [28]. Synonymized with *D. rufipes* by Sormova et al. [155].

### 3.3. Identification Key to Species of Genus Malacogaster Bassi, 1834 (Based on Males)

1. Clypeus distinctly produced forwards, with anterior margin rounded; labrum covered by clypeus (Figure 20e,f); Libya ………………………………………………………… *M. ruficollis* Dodero

–. Clypeus short, wide, with anterior margin concave; labrum fully exposed (e.g., Figure 9e) ………………………………………………………………………………………………………………………………… 2

2. Pronotum black; body more than 3.60 times as long as wide; combined elytra more than 2.50 times as long as wide (Figure 8a,g) ……………………………………*M. holomelas* Peyerimhoff

–. Pronotum yellowish to reddish brown; body 2.40–3.20 times as long as wide; combined elytra 1.55–2.25 times as long as wide ………………………………………………………………………….. 3

3. Head brown, medially reddish light brown, antennae yellowish brown to brown; paramere is apically narrowly rounded in lateral view (Figure 24a–h,l–n); The Canary Islands ……………………………………………………………………………………………………….. *M. tilloides* Wollaston

–. Head and most antennomeres dark brown to black; paramere is apically truncated in lateral view (e.g., Figure 5g) …………………………………………………………………………………………. 4

4. Femur and tibia uniformly yellowish brown to reddish brown ………………………………… 5 

–. Femur and tibia either uniformly dark brown to black or with only at most apical half of tibia lighter ……………………………………………………………………………………………………………….. 6

5. Head including eyes wider than pronotum when measured at widest place (Figure 19a,g); minimal interocular frontal separation less than twice maximum eye diameter; pronotum 1.15–1.20 times as wide as long when measured at widest place, widest medially or only slightly posteriorly (Figure 19g) ……………………………………. *M. rubripes* Peyerimhoff

–. Head including eyes is narrower than pronotum when measured at widest place (Figure 21c,g); minimal interocular frontal separation more than twice maximum eye diameter; pronotum 1.25 times as wide as long when measured at widest place, distinctly gradually widened posteriorly (Figure 21g) …………………………………… *M. rutllanti* Pardo Alcaide

6. Eyes are moderately large to large, their minimal frontal separation 1.85–2.30 times the maximum eye diameter; pronotum width at posterior angles 1.00–1.12 times the pronotum width at anterior angles …………………………………………………………………………………………. 7

–. Eyes are relatively small, their minimal frontal separation 2.40–2.95 times maximum eye diameter; pronotum width at posterior angles 1.15–1.30 times the pronotum width at anterior angles ………………………………………………………………………………………………………………….. 8

7. Pronotum is widest medially, both medially and posteriorly, or only slightly posteriorly; pronotum width at posterior angles 1.00–1.10 times as pronotum width at anterior angles; sides of pronotum rounded to subparallel-sided (Figure 9f, Figure 10e and Figure 11e) ……………………………………………………………………………………………………. *M. maculiventris* Reitter

–. Pronotum is clearly widest posteriorly; pronotum width at posterior angles 1.12 times as pronotum width at anterior angles; sides of pronotum are clearly concave (Figure 22a,e) …………………………………………………………………………………………………………………….. *M. theyri* Pic

8. Pubescence on apical half of elytra is yellowish to reddish brown; tibia either uniformly dark brown to black or with its apical half lighter, yellowish brown …… *M. passerinii* Bassi

–. Pubescence on apical half of elytra is reddish dark brown to black; tibia uniformly dark brown to black ………………………………………………………………………………. *M. nigripes* Schaufuss

## 4. Discussion

The genus *Malacogaster* has always been a stable member of Drilini (or Drilidae) and supposedly closely related to *Drilus* (e.g., [1,3,4,9,10,25]). In the most comprehensive molecular phylogeny of Drilini to date, Kundrata and Bocak [1] showed that *Malacogaster* falls within the “clade D”, together with other Palearctic genera *Drilus*, *Malacodrilus* Kundrata and Bocak, 2019 and *Drilorhinus* Kovalev et al., 2019. All included genera share similar morphology of adult males, including the serrate to pectinate antennae, relatively small eyes, with their minimum frontal separation 1.60–3.00 times the maximum eye diameter, the pronotum without sublateral carinae, the prosternum with a reduced prosternal process, the mesoventrite v-shaped with a reduced mesoventral process, elytra often divergent or shortened, and with a rough surface, and the abdomen with free ventrites [1]. Kundrata and Bocak [1] found the *Malacogaster* sister to monotypic *Malacodrilus* from Pakistan, which were both together sister to *Drilus*. In molecular phylogenetic analyses which did not contain *Malacodrilus* (nor *Drilorhinus*), *Malacogaster* was sister to *Drilus* [17,147], or even found to be a terminal lineage within the *Drilus* clade [11; partly also 1]. A close relationship between *Malacogaster* and *Drilus* was also confirmed by their similar micromorphology of antennae [16]. It should be noted that within the “(*Malacogaster* + *Malacodrilus*) + *Drilus*” clade in the preferred tree of Kundrata and Bocak [1], the monophyly of *Drilus* was not statistically supported, and in another analysis, “*Malacogaster* + *Malacodrilus*” subclade was found inside the *Drilus* clade. Therefore, the relationships among the genera of the “clade D” need further investigation.

*Malacogaster* differs from other genera in the “clade D” in having a lateral pronotal carina short, usually reaching no more than a half of the pronotal length (vs. lateral carina almost complete in *Drilus* and *Malacodrilus*, but incomplete, distinct at basal half, then only vaguely defined and missing after three quarters of the prothorax length in *Drilorhinus*). There were several additional characters previously used for the recognition of *Malacogaster*; however, this study showed that they can only be partly used. Most *Malacogaster* species have a short and wide fronto-clypeal region; however, *M. ruficollis* has a fronto-clypeal region produced and covering the labrum. Most *Malacogaster* species also have comparatively small eyes; however, specimens of *M. maculiventris*, *M. rubripes*, and *M. theyri* have eyes larger, and distinctly protruding. The pronotum shape in most *Malacogaster* species is somewhat characteristic for the genus—not transverse such as in *Drilus*, but more or less distinctly widened posteriorly, and with sides more or less straight or concave (Figure 4a). However, there are some exceptions; the pronotum of *M. ruficollis* is more transverse and somewhat constricted near the anterior angles (Figure 20g), and the pronotum of *M. tilloides* is usually widest anteriorly (in several cases medially) and with distinctly rounded sides (Figure 24h). One of the most prominent characters often used for the definition of *Malacogaster* are relatively short, posteriorly dehiscent elytra which do not fully cover the abdomen. Indeed, the abdominal segments of *Malacogaster* are connected by extensive membranes so that the whole abdomen is highly flexible, and usually several abdominal segments are surpassing the tip of the elytra (e.g., Figure 5a,b and Figure 18). The vast majority of *Malacogaster* have the combined elytra less or about twice as long as wide. On the other hand, *M. tilloides* has elytra up to 2.25 times as long as wide (Figure 23 and Figure 24a), and especially *M. holomelas* have relatively elongated elytra, which are more than 2.50 times as long as wide (Figure 8a).

An additional prominent character of *Malacogaster* is the elongated and subparallel-sided abdominal sternite IX, which is usually 2.15–2.70 times as long as wide (Figure 5e). However, *M. ruficollis* has sternite IX only 1.65 times as long as wide, and with clearly rounded sides (Figure 20j). Abdominal tergite X in *Malacogaster* is usually also elongated, 1.85–2.25 times as long as wide; however, it is only 1.50 times as long as wide in *M. tilloides*, and subquadrate, 0.95 times as long as wide in *M. ruficollis* (Figure 5c, Figure 20h and Figure 24j). Tergite X of the latter species resembles in the relative length and shape those of the genus *Drilus* [e.g., 138]. The last character previously thought to be characteristic for *Malacogaster* is the apically truncate paramere with a latero-apical projection on an inner side [1] (Figure 5g). However, the parameres in *M. ruficollis* and *M. tilloides* are not truncate apically (clearly visible from the lateral view) and do not bear any distinct projection on an inner side (Figure 20k–m and Figure 24l–n). They apparently remind the parameres found in *Drilus*.

It should also be noted, that the body coloration of vast majority of species is very similar, with dark brown to black head and elytra, and a yellowish to reddish brown pronotum. This probably also played a role in assigning the species in *Malacogaster* by early authors, sometimes wrongly as in the case of *M. akbesiana* from Asia Minor [27,57]. However, there is a single species with a black pronotum, i.e., *M. holomelas* from Morocco (Figure 8g), and *M. tilloides* from the Canary Islands that has a relatively pale head (Figure 24h). Additionally, earlier authors identified some species of *Malacogaster* mainly based on coloration. However, since coloration in some Drilini species can be variable [13,155], it is not surprising that *Malacogaster* species also show some degree of variability in the coloration of certain parts such as the antennae, legs, and basal abdominal ventrites. The darker coloration of basal abdominal ventrites was used for the definition of *M. maculiventris*; however, this character is highly unstable and does not correspond with other morphological features. There are some specimens of *M. maculiventris* which have abdomen almost completely light reddish brown or with only some traces of dark coloration in basal abdominal ventrites. On the other hand, there are some other species, such as *M. passerinii*, in which several specimens have the basal part of the abdomen distinctly darker. Therefore, the coloration in *Malacogaster* should be treated with caution. 

Based on the above-mentioned variability in *Malacogaster*, we can conclude that *M. ruficollis* from Libya is morphologically the most distinct species in the genus (e.g., clypeus distinctly produced forwards and covering labrum, unique shape of pronotum, pregenital segments and genitalia like in *Drilus*), followed by *M. tilloides* from the Canary Islands (pale head, relatively long elytra, unique shape of pronotum, paramere like in *Drilus*). Another distinctive species is *M. holomelas* from Morocco, which has a black pronotum and elongated elytra. However, its male pregenital segments and genitalia are typical for *Malacogaster*. All remaining species are superficially very similar (head and elytra dark brown to black, pronotum yellowish to reddish brown, elytra relatively short, exposing several abdominal segments, elongate parallel-sided sternite IX and tergite X, and apically truncate parameres with a latero-apical protrusion) and mostly differ in coloration of legs and hairs, the relative size of eyes, and the shape of the pronotum.

Besides the missing phylogenetic analysis which would probably help us understand the identity of some species and solve the situation in the *passerinii*/*nigripes* complex, we are also missing information on the morphology, biology, and ecology of immature stages in most species of *Malacogaster*, as well as on the females. Although several studies provided information on immature stages, there are problems with species identifications of the studied specimens (e.g., [4,38,48]). Additionally, we have no comparative study on females within the genus, as we simply do not know females for most species. Since they are paedomorphic and remain larviform during their adulthood, similarly as in other groups of Elateroidea [3,165,166,167,168], their assignment to a species-level is usually complicated or even impossible as they dramatically differ in morphology from their counterparts (Figure 2 and Figure 6). Recent research on *Drilus* in the Mediterranean showed that the DNA barcoding approach is a powerful tool for matching larvae, females and males in Drilini [139,155]. This approach should also be used for *Malacogaster*.

## Data Availability

Not applicable.
